# Supplementary material for: Bright, efficient, and stable pure-green hyperfluorescent organic light-emitting diodes by judicious molecular design
Source: Nat Commun. 2024 Apr 12;15:3174. doi: 10.1038/s41467-024-47482-3 (PMC11014922; doi:10.1038/s41467-024-47482-3)
Supplement: Supplementary file 1 — Supplementary Information [file 41467_2024_47482_MOESM1_ESM.pdf]

Supplementary Information

Bright, Efficient, and Stable Pure-Green Hyperfluorescent Organic Light-Emitting Diodes by Judicious Molecular Design

Yi-Ting Lee[1,2], Chin-Yiu Chan[3,4,*], Nanami Matsuno[5], Shigetada Uemura[5], Susumu Oda[6], Masakazu Kondo[7], Rangani Wathsala Weerasinghe[1], Yanmei Hu[1], Gerardus N. Iswara Lestanto[1], Youichi Tsuchiya[1], Yufang Li[3,4], Takuji Hatakeyama[5,*] , and Chihaya Adachi[1,8,*]

[1]*Center for Organic Photonics and Electronics Research (OPERA), Kyushu University, Motooka, Nishi, Fukuoka 819-0395, Japan.*

[2]*Department of Chemistry, Soochow University, 70 Linhsi Road, Shihlin District, Taipei, Taiwan 111002, R.O.C.*

[3]*Department of Materials Science and Engineering, City University of Hong Kong, Tat Chee Avenue, Kowloon, Hong Kong SAR, China.*

[4]*Department of Chemistry, City University of Hong Kong, Tat Chee Avenue, Kowloon, Hong Kong SAR, China.*

[5]*Department of Chemistry, Graduate School of Science, Kyoto University, Sakyo-ku, Kyoto 606-8502, Japan.*

[6]*Department of Applied Chemistry, Graduate School of Science and Engineering, Toyo University, 2100 Kujirai, Kawagoe, Saitama 350-8585, Japan.*

[7]*JNC Co., Ltd. 5-1 Goikaigan, Ichihara, Chiba 290-8551, Japan*

[8]*International Institute for Carbon Neutral Energy Research (WPI-I2CNER), Kyushu University, 744 Motooka, Nishi, Fukuoka 819-0395, Japan.*

E-mail: chinychan2@cityu.edu.hk, hatake@kuchem.kyoto-u.ac.jp, adachi@cstf.kyushu-u.ac.jp

Supplementary Table 1. Summary of device performance of green MREs in the literature.

Emitter	Host	λ_{em} (nm)[a]	FWHM (nm)[b]	CIE (x,y)[c]	V_{on} (V)[d]	EQE (%)[e]	CE_{max} (cd A^{-1})[f]	PE_{max} (lm W^{-1})[g]	LT (h)[h]	Ref.
**ω-DABNA** (0.5 wt%) Device A	DOBNA-Ph	512	25	(0.13, 0.73)	3.0	30.8/ 30.4/ 29.2	101.9	106.2	LT95 = 34 @ 1000 nits	This work
**ω-DABNA-M** (0.5 wt%) Device B	DOBNA-Ph	515	25	(0.15, 0.74)	3.1	32.7/ 32.0/ 27.4	114.1	114.5	LT95 = 24 @ 1000	This work
**ω-DABNA-PH** (0.5 wt%) Device C	DOBNA-Ph	521	30	(0.19, 0.74)	3.2	31.8/ 30.5/ 27.4	123.6	124.8	LT95 = 27 @ 1000 nits	This work
**ω-DABNA** (0.5 wt%) Device D	mCBP: 3Cz2DPhCzBN [I]	511	24	(0.15, 0.64)	3.4	28.0/ 25.1/ 23.9	82.5	86.4	LT95 = 188 @ 1000 nits	This work
**ω-DABNA-M** (0.5 wt%) Device E	mCBP: 3Cz2DPhCzBN [I]	515	25	(0.20, 0.66)	3.0	28.3/ 26.8/ 25.0	86.4	90.5	LT95 = 230 @ 1000 nits	This work
**ω-DABNA-PH** (0.5 wt%) Device F	mCBP: 3Cz2DPhCzBN [I]	521	30	(0.18, 0.65)	3.0	27.3/ 25.6/ 24.6	89.6	93.8	LT95 = 205@ 1000 nits	This work
**ω-DABNA** (1 wt%) Device G	mCBP: 3Cz2DPhCzBN [I]	513	23	(0.14, 0.69)	3.5	26.8/ 22.3/ 21.2	76.6	75.2	LT95 = 143 @ 1000 nits	This work
**ω-DABNA-M** (1 wt%) Device H	mCBP: 3Cz2DPhCzBN [I]	517	23	(0.17, 0.70)	3.0	28.2/ 25.3/ 22.6	94.2	98.7	LT95 = 110 @ 1000 nits	This work
**ω-DABNA-PH** (1 wt%) Device I	mCBP: 3Cz2DPhCzBN [I]	522	29	(0.21, 0.69)	3.0	26.5/ 24.9/ 23.1	94.2	98.6	LT95 = 109 @ 1000 nits	This work
**ω-DABNA-PH** (1 wt%) Device J	mCBP: 4CzIPN [I]	522	30	(0.24, 0.70)	2.6	24.4/ 21.7/ 18.5	89.0	107.5	LT95 = 640 @ 1000 nits	This work
**ν-DABNA-CN-Me**	DOBNA-Ph	504	23	(0.13, 0.65)	3.0	31.9/ 31.5/ 28.5	88.9	93.6	LT80 = 59 @ 500 nits	1
**2F-BN**	mCPCB: 5TCzBN [I]	501	40	(0.16, 0.60)	2.6	22.0/ 20.1/ 15.0	-	69.8	LT90= 45.8 @ 2000 nits	2
**AZA-BN**	mCBP:$Ir(ppy)_3$ [I]	527	30	(0.27, 0.69)	2.6	28.2/ 26.5/ 19.1	-	121.7	LT90 = 46.3 @ 2000 nits	3

**m-Cz-BNCz**	PhCzBCz	520	44	(0.23, 0.69)	3.0	27.0/ 24.2/ 14.4	99.2	103.9	-	4
**(*R*)-OBN-2CN-BN**	PhCzBCz: 5CzBN [I]	496	33	(0.13, 0.53)	2.8	29.8/ 27.2/ 21.2	71.9	79.8	-	5
**BN-DPAC**	mCBP	504	48	(0.14, 0.56)	3.5	28.2/ 27.7/ 19.2	77.3	56.5	-	6
**2PTZBN**	mCBP:PO-T2T	528	58	(0.28, 0.65)	3.0	25.5/ 21.7/ 17.2	96.5	86.6	-	7
**BN-1**	mCBP:PO-T2T	506	36	(0.15, 0.63)	3.0	24.3/ 18.4/ 12.9	72.8	65.3	-	8
**PXN-BN**	mCBP	516	47	(0.22, 0.67)	3.6	23.3/ 19.9/ 11.3	83.8	70.1	-	9
**OAB-ABP-1**	Polymer B	505	33	(0.12, 0.63)	3.5	21.8/ 19.6/ 17.4	53.2	45.3	LT50 = 11 @ 300 nits	10
**CzB2-M/P**	DOBNA-Tol	497	29	(0.12, 0.57)	3.4	26.7/ 24.4/ 18.0	67.8	62.6	LT80 = 74 @ 500 nits	11
**BBCz-G**	mCBP	515	54	(0.26, 0.68)	-	31.8/-/-	-	-	-	12
**(–)-BN5**	mCPCN	506	48	(0.17, 0.60)	4.8	26.5/ 17.6/ 11.1	79.4	44.5	-	13
**BN-TP**	PhCzBCz	528	36	(0.26, 0.70)	3.1	35.1/ 32.4/ 20.8	139.3	139.3	LT50 = 28.8 @ 4000 nits	14
**2Cz-PTZ-BN**	PhCzBCz	516	56	(0.24, 0.63)	3.3	32.8/ 30.8/ 23.5	108.5	92.1	LT50 = 14.1@ 500 nits	15
**(R)-BN-MeIAc**	DMIC-TRZ	504	33	(0.12, 0.63)	2.4	37.2/ 36.1/ 26.1	103.0	130.2	LT50 = 16.3 @ 1000 nits	16
**DBNO**	PhCzBCz: 5TCzBN [I]	504	27	(0.14, 0.53)	3.0	37.1/ 30.8/ 20.6	100.1	105.6	LT50 = 2.9 @ 500 nits	17
**DtBuCzB**	mCBP: CTPCF3 [I]	488	29	(0.13, 0.40)	3.0	27.5/ 26.9/ 24.1	-	-	LT90 =60.2 @ 500 nits	18

[a]Maximum wavelength of EL spectrum. [b]Full-width at half-maximum. [c]CIE(x,y) coordinates. [d]Turn-on voltages at 1 cd m^{-2}. [e]External quantum efficiencies: maximum value at 1 cd m^{-2}, value at 100 cd m^{-2} and value at 1000 cd m^{-2}. [f]Maximum current efficiency. [g]Maximum power efficiency. [h]Lifetime. [I]Sensitizer.

Supplementary Note 1

All reagents were used as received from commercial sources and were used without further purification. Chromatographic separations were carried out using silica gel (200–300 nm). The two materials investigated in this paper were synthesized by following the procedures described below. All compounds were purified twice by temperature gradient vacuum sublimation. ^{1}H, ^{11}B and ^{13}C nuclear magnetic resonance (NMR) spectra were with a Bruker Biospin Avance-III 500 NMR spectrometer at ambient temperature. Chemical shifts (δ) are given in parts per million (ppm) relative to tetramethylsilane (TMS; δ = 0) as the internal reference. High-resolution mass spectra were measured in MALDI-TOF on a Waters 3100 mass detector. Elemental analyses (C, H and N) were carried out with a Yanaco MT-5 elemental analyser. **ω-DABNA** was synthesized according to the literature.[19]

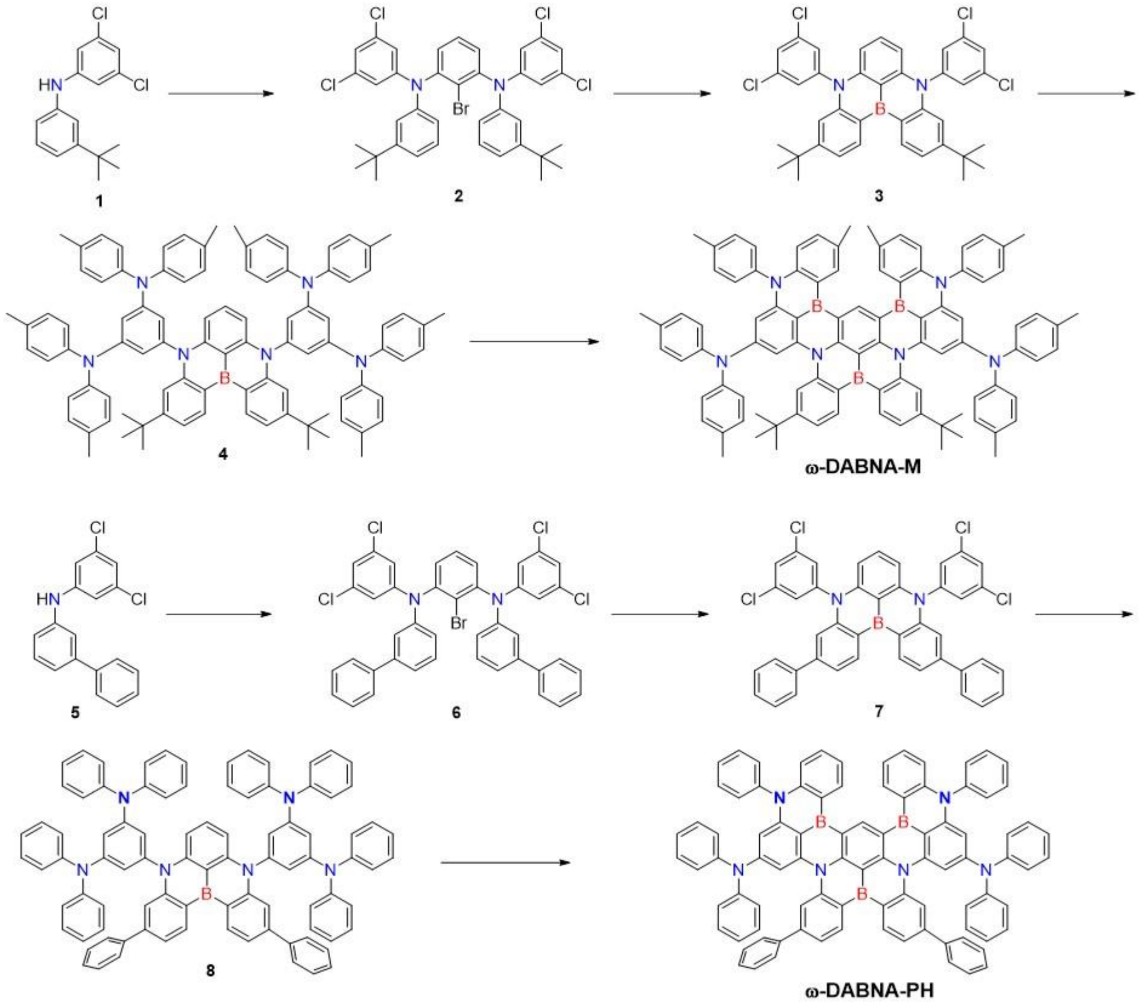

Supplementary Figure 1. Synthetic route for two new MREs.

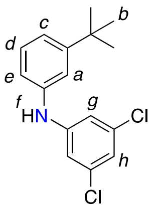

N-[3-(*tert*-butyl)phenyl]-3,5-dichloroaniline (1).Tris(dibenzylideneacetone)dipalladium(0) (0.740 g, 0.80 mmol), 2,2′-bis(diphenylphosphino)-1,1′-binaphthyl (1.00 g, 1.6 mmol), 3,5-dichloroaniline (6.48 g, 40 mmol), 1-bromo-3-*tert*-butylbenzene (9.40 g, 44 mmol), and sodium *tert*-butoxide (4.62 g, 48 mmol) were dissolved in toluene (300 mL) under a nitrogen atmosphere. After stirring at 100 °C for 18 h, the reaction mixture was allowed to cool to room temperature. After addition of 1 N hydrochloric acid (230 mL) to the reaction mixture, the aqueous layer was extracted with toluene (100 mL, three times). The combined organic layers were removed *in vacuo*. The crude product was purified by silica gel column chromatography (eluent: hexane, hexane/toluene = 3/1) to obtain the title compound (10.7 g, 91% yield, >98% pure on NMR analysis) as a yellow oil. Analytical data for the title compound have been reported.[19]

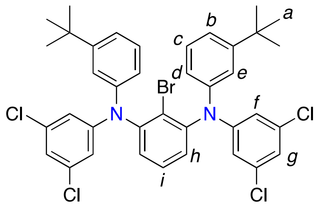

2-Bromo-*N*^{1},*N*^{3}-bis[3-(*tert*-butyl)phenyl]-*N*^{1},*N*^{3}-bis(3,5-dichlorophenyl)benzene-1,3-diamine (2). Tris(dibenzylideneacetone)dipalladium(0) (1.10 g, 1.2 mmol), 1,2,3-tribromobenzene (6.30 g, 20 mmol), tri-*tert*-butylphosphonium tetrafluoroborate (1.39 mg, 4.8 mmol), and sodium *tert*-butoxide (11.5 g, 120 mmol) were dissolved in **1** (14.1 g, 48 mmol), and toluene (200 mL) under a nitrogen atmosphere. After stirring at 90 °C for 16 h, the reaction mixture was allowed to cool to room temperature. After the reaction mixture was filtered with a pad of silica gel (eluent: toluene), the solvent was removed *in vacuo*. The crude product was purified by silica gel column chromatography (eluent: hexane, hexane/toluene = 10/1) to obtain the title compound (8.84 g, 60% yield, 99% pure on NMR analysis) as a white solid. Analytical data for the title compound have been reported.[19]

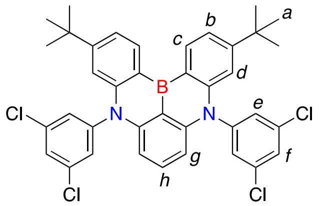

3,11-Di-*tert*-butyl-5,9-bis(3,5-dichlorophenyl)-5,9-dihydro-5,9-diaza-13b-bora naphtho[3,2,1-*de*]anthracene (3). A solution of butyllithium in hexane (7.75 mL, 1.59 M, 12 mmol) was added slowly to **2** (6.06 g, 8.2 mmol) in toluene (40 mL) at –78 °C under a nitrogen atmosphere. After stirring at 0 °C for 2 h, boron tribromide (1.58 mL, 16 mmol) was added at 0 °C. After stirring at 50 °C for 14 h, reaction mixture was diluted by dichloromethane (20 mL) and quenched by phosphate buffer solution (pH 6.8, 100 mL) at 0 °C. The aqueous layer was separated and extracted with dichloromethane (100 mL, three times). After the solvent was removed in *vacuo*, the crude product was washed with methanol to obtain the title compound (2.34 g, 42% yield, 97% pure on NMR analysis) as a yellow solid. Analytical data for the title compound have been reported.[19]

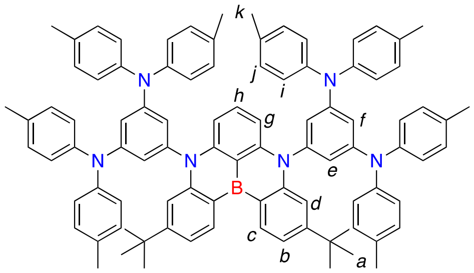

5,5'-(3,11-Di-*tert*-butyl-5,9-diaza-13b-boranaphtho[3,2,1-*de*]anthracene-5,9-diyl)bis(N^1,N^1,N^3,N^3-tetra-*p*-tolylbenzene-1,3-diamine) (4). Tris(dibenzylideneacetone)dipalladium(0) (27.5 mg, 0.030 mmol), **3** (0.335 g, 0.50 mmol), di-*p*-tolylamine (0.560 g, 3.0 mmol), tri-*tert*-butylphosphonium tetrafluoroborate (35.1 mg, 0.12 mmol) and sodium *tert*-butoxide (0.289 g, 3.0 mmol) were dissolved in *o*-xylene (5.0 mL) under a nitrogen atmosphere. After stirring at 120 °C for 18 h, the reaction mixture was allowed to cool to room temperature. After the reaction mixture was filtered with a pad of silica gel (eluent: toluene), the solvent was removed *in vacuo*. After the crude product was purified by washing with methanol (60 mL) and silica gel column chromatography (eluent: hexane/toluene = 2/1, two times) to obtain the title compound (0.542 g, 82% yield, 98% pure on ^{1}H NMR analysis) as a yellow solid. IR (neat): cm^{-1} 2955, 2858, 2963, 1607, 1587, 1568, 1504, 1440, 1418, 1361, 1315, 1439, 1308, 1284, 1248, 1229, 1184, 1173, 1148, 1036, 993, 970, 862, 812, 787, 727, 710, 694, 658; mp: 254.6–250.3 °C; ^{1}H

NMR (500 MHz, $CDCl_3$) δ 1.39 (s, 18H, *a*), 2.23 (s, 24H, *k*), 6.47 (d, *J* = 8.2 Hz, 2H, *g*), 6.52 (s, 4H, *e*), 6.93 (s, 2H, *f*), 6.95–7.00 (m, 34H, *d, i, j*), 7.23 (d, *J* = 8.2 Hz, 2H, *b*), 7.47 (t, *J* = 8.2 Hz, 1H, *h*), 8.72 (d, *J* = 8.2 Hz, 2H, *c*); $^{13}C\{^1H\}$ NMR (124 MHz, $CDCl_3$) δ 20.7 (6C), 31.2 (8C), 30.1 (2C), 104.6 (2C), 113.7 (2C), 117.0 (2C), 117.2 (2C), 117.8 (4C), 121.5 (2C), 124.0 (1C), 124.3 (16C), 129.8 (16C), 131.6 (1C), 132.5 (8C), 134.5 (2C), 143.7 (2C), 144.8 (8C), 146.4 (2C), 147.2 (2C), 151.1 (4C), 153.4 (2C); ^{11}B NMR (159 MHz, $CDCl_3$) δ 40.5.

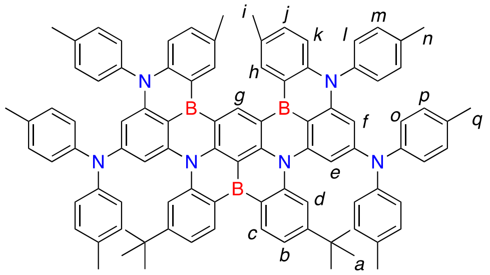

3,23-Di-*tert*-butyl-11,15-dimethyl-*N*^{6},*N*^{6},*N*^{20},*N*^{20},8,18-hexa-*p*-tolyl-8*H*, 18*H*-4b, 8,18, 21b-tetraaza-12b,13b,25b-triborabenzo[3,4]phenanthro[2,1,10,9-*qrst*]dinaphtho[3,2,1 -*de*:1',2',3'-*jk*]pentacene-6,20-diamine (ω-DABNA-M). Boron tribromide (0.308 mL, 3.2 mmol) was added to a solution of **5** (0.240 g, 0.20 mmol) and 2,6-di-*tert*-butylpyridine (0.260 mL, 1.2 mmol) in toluene (2.0 mL) under a nitrogen atmosphere. After stirring at 120 °C for 24 h, reaction mixture was diluted by dichloromethane (10 mL) and quenched by phosphate buffer solution (pH 6.8, 30mL) at 0 °C. The aqueous layer was separated and extracted with dichloromethane (50 mL, three times). After the combined organic layer was condensed *in vacuo*, the yield of the title compound in the crude product was determined to be 49% yield by ^1H NMR analysis using dibromomethane as an internal standard. The crude product was purified by silica gel column chromatography (eluent: hexane/dichloromethane = 3/2) to obtain the title compound (93.0 mg, 35% yield, 98% pure on ^1H NMR analysis) as a yellow solid. IR(neat): cm^{-1} 2955, 2862, 1748, 1717, 1607, 1589, 1558, 1541, 1506, 1373, 1361, 1314, 1296, 1248, 1234, 1217, 1184, 1170, 1138, 1103, 1036, 978, 951, 796, 729, 559, 550; mp: >400 °C; ^1H NMR (500 MHz, $CDCl_3$) δ 1.27 (s, 18H, *a*), 2.26 (s, 12H, *q*), 2.41 (s, 6H, *i*), 2.43 (s, 6H, *n*), 5.85 (s, 2H, *f*), 6.77 (d, *J* = 8.8 Hz, 2H, *k*), 6.87 (d, *J* = 8.2 Hz, 8H, *o*), 6.99 (d, *J* = 8.2 Hz, 8H, *p*), 7.14 (s, 2H, *e*), 7.25–7.29 (m, 10H, *j, l, m*), 7.37 (d, *J* = 7.9 Hz, 2H, *b*), 8.32 (s, 2H, *d*), 8.49 (d, *J* = 7.9 Hz, 2H, *c*), 8.68 (s, 2H, *h*), 10.3 (s, 1H, *g*); $^{13}C\{^1H\}$ NMR (124 MHz, $CDCl_3$) δ 20.80 (4C), 20.81 (2C), 21.2 (2C), 31.1 (6C), 35.2 (2C), 103.6 (2C), 106.6 (4C), 117.0 (2C), 117.7 (1C), 119.2 (2C), 119.7 (2C), 121.0 (2C), 124.3 (2C), 125.1 (8C), 127.4 (2C), 129.1 (2C), 129.7 (8C), 130.2 (1C), 130.9 (2C), 131.3 (2C), 132.1 (4C), 132.6 (4C), 133.7 (2C), 135.1

(2C), 137.9 (2C), 139.4 (2C), 144.7 (4C), 146.2 (2C), 147.1 (2C), 147.4 (2C), 147.8 (2C), 148.0 (2C), 149.4 (2C), 150.4 (2C), 152.5 (2C); ^{11}B NMR (159 MHz, $CDCl_3$) δ 39.4; HRMS (MALDI-TOF/MS) *m/z* $[M]^+$ calcd for $C_{94}H_{83}B_3N_6$ 1328.6989; observed 1328.7000.

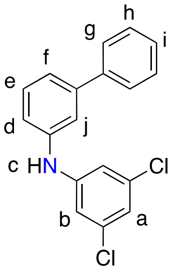

***N*-(3,5-Dichlorophenyl)-[1,1'-biphenyl]-3-amine (5).** Tris(dibenzylideneacetone)dipalladium(0) (0.733 g, 0.80 mmol), 2,2'-bis(diphenylphosphino)-1,1'-binaphthyl (1.00 g, 1.6 mmol), 3,5-dichloroaniline (6.48, 40 mmol), 3-bromo-1,1'-biphenyl (9.37 g, 44 mmol), and sodium *tert*-butoxide (4.61 g, 48 mmol) were dissolved in toluene (300 mL) under a nitrogen atmosphere. After stirring at 90 ºC for 18 h, the reaction mixture was allowed to cool to room temperature. After addition of 1 N hydrochloric acid (100 mL) to the reaction mixture, the aqueous layer was extracted with toluene (200 mL, three times). The combined organic layers were removed *in vacuo*. The crude product was purified by silica gel column chromatography (eluent: hexane, hexane/toluene = 4/1) to obtain the title compound (10.7 g, 91% yield, >98% pure on NMR analysis) as a white solid. IR(neat): cm^{-1} 3383 (N-H), 2359, 2341, 1581, 1568,1477, 1445, 1315, 1080, 1032, 957, 831, 806, 756, 709, 694, 667; mp: 87.2–88.5 °C; ^{1}H NMR (400 MHz, $CDCl_3$) δ 5.77 (s, 1H, *c*), 6.85 (s, 1H, *a*), 6.88 (s, 2H, *b*), 7.07–7.09 (d, *J* = 7.5 Hz, 1H, *f*), 7.27–7.28 (m, 2H, *d, j*), 7.34–7.39 (m, 2H, *e, i*), 7.42–7.45 (d, *J* = 7.6 Hz, 2H, *h*), 7.55–7.56 (d, *J* = 6.9 Hz, 2H, *g*); ^{13}C NMR (101 MHz, $CDCl_3$) δ 114.9 (2C), 119.0 (1C), 119.0 (1C), 120.2 (1C), 122.2 (1C), 127.2 (2C), 127.7 (1C), 128.9 (2C), 130.1 (1C), 135.7 (2C), 140.7 (1C), 141.4 (1C), 143.0 (1C), 145.9 (1C).

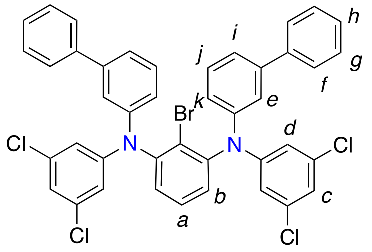

***N*¹,*N*³-Di((1,1'-biphenyl)-3-yl)-2-bromo-*N*¹,*N*³-bis(3,5-dichlorophenyl)benzene-1,3-diamine (6).** Tris(dibenzylideneacetone)dipalladium(0) (2.47 g, 2.7 mmol), 1,2,3-tribromobenzene (14.2 g, 45 mmol), tri-*tert*-butylphosphonium tetrafluoroborate (3.13 g, 11 mmol), and sodium *tert*-butoxide (26.0 g, 270 mmol) , and **5** (34.0 g, 110 mmol) were dissolved in toluene (350 mL) under a nitrogen atmosphere. After stirring at 90 ºC for 20 h, the reaction mixture was allowed to cool to room temperature. After addition of 1 N hydrochloric acid (100 mL) to the reaction mixture, the aqueous layer was extracted with toluene (200 mL, three times). The combined organic layers were removed *in vacuo*. The crude product was purified by washing with methanol (1000 mL) and silica gel column chromatography (eluent: hexane, hexane/toluene = 3/1) and washing with methanol (300 mL) and washing with hexane (150 mL) to obtain the title compound (17.5 g, 50% yield, 99% pure on NMR analysis) as a white solid. IR(neat): cm^{-1} 2924, 2853, 1580, 1562, 1558,1476, 1456, 1441, 1416, 1310, 1296, 1273, 1233, 1111,1096, 1055, 1040, 988, 831, 800, 754, 732, 716, 698, 675, 754; mp: 199.7–200.6 °C; ^{1}H NMR (400 MHz, $CDCl_3$) δ 6.76 (s, 4H, *d*), 6.91 (s, 2H, *c*), 7.04–7.06 (d, *J* = 7.3 Hz, 2H, *b*), 7.21–7.23 (d, *J* = 7.8 Hz, 2H, *k*), 7.30 (s, 2H, *e*), 7.32–7.36 (m, 5H, *a, h, j*), 7.37–7.43 (m, 6H, *g, i*), 7.49–7.51 (d, *J* = 7.3 Hz, 4H, *f*); ^{13}C NMR (101 MHz, $CDCl_3$) δ 118.4 (4C), 121.4 (2C), 122.6 (2C), 122.7 (2C), 123.3 (2C), 125.5 (1C), 127.2 (4C), 127.7 (2C), 128.9 (4C), 129.7 (2C), 130.1 (2C), 130.3 (1C), 135.6 (4C), 140.5 (2C), 142.9 (2C), 145.9 (2C), 146.9 (2C), 148.9 (2C).

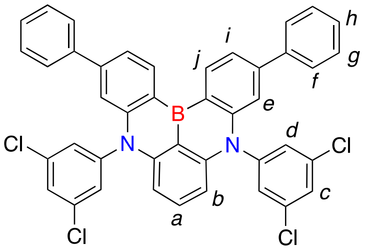

Bis(3,5-dichlorophenyl)-3,11-di-*tert*-butyl-5,9-dihydro-5,9-diaza-13b-boranaphtho[3,2,1-de]anthracene (7). A solution of butyllithium in hexane (3.03 mL, 1.65 M, 5.0 mmol) was added slowly to **6** (3.10 g, 4.0 mmol) in toluene (20 mL) at –78 ºC under a nitrogen atmosphere. After stirring at 0 ºC for 2 h, boron tribromide (0.759 mL, 8.0 mmol) was added at 0 ºC. After stirring at 50 °C for 18 h, reaction mixture was diluted by dichloromethane (10 mL) and quenched by phosphate buffer solution (pH 6.8, 50 mL) at 0 °C. The aqueous layer was separated and extracted with dichloromethane (100 mL, three times). After the solvent was removed in *vacuo*, the crude product was washed with methanol (1500 mL) to obtain the title compound (1.43 g, 50% yield, 97% pure on NMR analysis) as a yellow solid. IR(neat): cm^{-1} 3078, 2924, 2852, 1713, 1582, 1564, 1503, 1479, 1456, 1439, 1408, 1312, 1296,

1273, 1242, 1234, 1184, 1165, 1111, 1076, 1055, 1043, 1026, 980, 912, 864, 856, 823, 802, 787, 756, 735, 719, 698, 678, 669, 654; mp: >300 °C; ^{1}H NMR (400 MHz, $CDCl_3$) δ 6.18–6.20 (d, *J* = 6.2 Hz, 2H, *b*), 6.94 (s, 2H, *c*), 7.37–7.41 (m, 7H, *a, d, i*), 7.45–7.49 (t, *J* = 7.6 Hz, 4H, *g*), 7.52–7.59 (m, 6H, *f, h*), 7.65 (s, 2H, *e*), 9.00–9.02 (d, *J* = 8.2 Hz, 2H, *j*); ^{13}C NMR (101 MHz, CD_2Cl_4) δ 105.9 (2C), 115.0 (2C), 117.3 (1C), 120.2 (2C), 123.0 (2C), 127.3 (1C), 127.6 (4C), 128.2 (2C), 129.2 (4C), 129.7 (4C), 132.8 (2C), 135.9 (1C), 137.4 (4C), 141.0 (2C), 143.9 (2C), 144.2 (2C), 146.4 (2C), 147.5 (2C); ^{11}B NMR (128 MHz, CD_2Cl_4) δ 41.1.

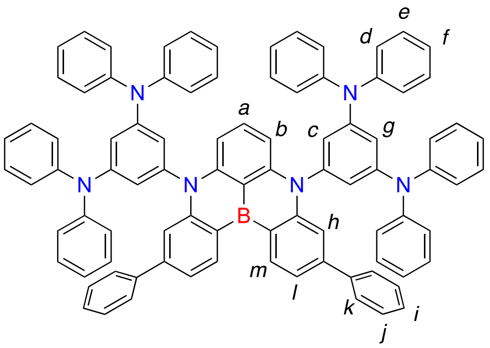

5,5'-(3,11-Diphenyl-5,9-diaza-13b-boranaphtho[3,2,1-de]anthracene-5,9-diyl)bis(N^1,N^1,N^3,N^3-tetraphenylbenzene-1,3-diamine) (8). Tris(dibenzylideneacetone)dipalladium(0) (0.00549 g, 0.0060 mmol), **7** (0.0710 g, 0.10 mmol), diphenylamine (0.102 g, 0.60 mmol), tri-*tert*-butylphosphonium tetrafluoroborate (0.693 g, 0.24 mmol) and sodium *tert*-butoxide (0.0577 g, 0.60 mol) were dissolved in *o*-xylene (1 mL) under a nitrogen atmosphere. After stirring at 120 °C for 16 h, the reaction mixture was allowed to cool to room temperature. After the reaction mixture was filtered with a pad of silica gel (eluent: toluene), the solvent was removed *in vacuo*. After the crude product was purified by silica gel column chromatography (eluent: hexane/toluene = 2/1, 1.5/1) and washing with hexane (10 mL, two times) and washing with acetonitrile (10 mL, three times) to obtain the title compound (0.043 g, 30% yield, 85% pure on HPLC analysis) as a yellow solid. IR (neat): cm^{-1} 1608, 1587, 1576, 1541, 1528, 1493, 1466, 1450, 1437, 1412, 1400, 1366, 1339, 1290, 1275, 1244, 1188, 1153, 1146, 1078, 1032, 937, 908, 895, 868, 814, 787, 764, 748, 712, 691, 679; mp: >300 °C; ^{1}H NMR (500 MHz, $CDCl_3$), δ 6.55–6.57 (d, *J* = 8.2 Hz, 2H, *b*), 6.67 (s, 4H, *c*), 6.91–6.95 (t, *J* = 6.6 Hz, 8H, *f*), 7.03 (s, 2H, *h*), 7.10–7.16 (m, 32H, *d, e*), 7.23 (s, 2H, *g*), 7.44–7.49 (m, 4H, *i, l*), 7.53–7.58 (m, 5H, *a, j*), 7.67–7.69 (d, *J* = 6.9 Hz, 4H, *k*), 8.86–8.88 (d, *J* = 8.2 Hz, 2H, *m*); ^{13}C NMR (101 MHz, $CDCl_3$) δ 105.2 (2C), 115.7 (2C), 117.4 (1C), 118.1 (2C), 119.0 (4C+2C), 123.0 (2C), 123.4 (8C), 124.4 (16C), 127.4 (4C), 127.7 (2C), 128.9 (4C), 129.4 (16C), 132.2 (1C), 135.5 (2C), 141.8 (2C), 143.4 (2C), 143.7 (2C), 146.4 (2C), 147.1 (8C), 147.6 (2C), 151.0 (4C); ^{11}B NMR (128 MHz, $CDCl_3$) δ 40.8.

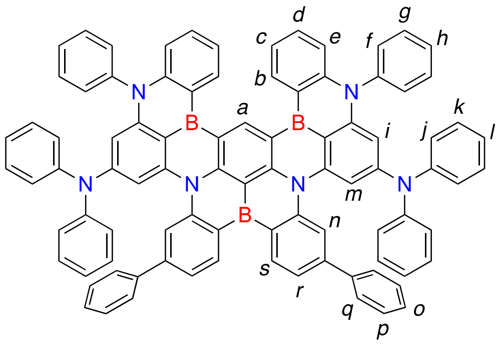

N^6,N^6,N^{20},N^{20},3,8,18,23-Octaphenyl-8*H*,18*H*-4b,8,18,21b-tetraaza-12b,13b,25b-triborabenzo[3,4]phenanthro[2,1,10,9-*qrst*]dinaphtho[3,2,1-*de*:1',2',3'-*jk*]pentacene-6,20-diamine (ω-DABNA-PH). Compound **9** (0.434 g, 0.35 mmol), boron triiodide (1.10 g, 2.8 mmol) and 2,6-di-*tert*-butylpyridine (0.402 g, 2.1 mmol) were dissolved in toluene (5 mL) at 0 °C under a nitrogen atmosphere. After stirring at 0 °C for 2 h, reaction mixture was diluted by dichloromethane (5 mL) and quenched by phosphate buffer solution (pH 6.8, 25 mL) at 0 °C. The aqueous layer was separated and extracted with dichloromethane (50 mL, three times). After the solvent was removed in *vacuo*, the crude product was separated into high and low polar components by silica gel column chromatography (eluent: hexane/toluene = 1/1, ethyl acetate). Acetic acid (2.0 mL, 35.0 mmol) was added to the high polar component in toluene (5.0 mL) at room temperature. After stirring at 80 ºC for 24 h, saturated sodium carbonate aqueous solution (20 mL) was added to the reaction mixture, and then the aqueous layer was extracted with dichloromethane (30 mL, three times). The combined organic layer was concentrated *in vacuo*. The yields of the title compound in the high and low polar components were determined to be 24 and 35% (total yield: 59%), respectively, by ^{1}H NMR analysis using 1,1,2,2-tetrachloroethane and dibromomethane as an internal standard. The resulting crude product was purified by silica gel column chromatography (eluent: hexane/toluene = 1/1) and washing with hexane (150 mL) and toluene (100 mL) to obtain the title compound (0.115 g, 95% pure on NMR analysis) as a yellow solid. IR(neat): cm^{-1} 1608,1576, 1539, 1504, 1493, 1437, 1402, 1387, 1321, 1292, 1267, 1248, 1213, 1182, 1163, 1138, 1078, 1067, 1030, 1003, 941, 908, 895, 868, 849, 827, 764, 750, 692, 658; mp: >300 °C; ^{1}H NMR (500 MHz, $CDCl_3$) δ5.86 (s, 2H, *i*), 6.77–6.83 (m, 12H, *i, l*), 6.86–6.90 (m, 10H, *e, k*), 7.16–7.18 (t, *J* = 6.2 Hz, 2H, *h*), 7.24–7.27 (t, *J* = 6.8 Hz, 2H, *c*), 7.30 (s, 2H, *m*), 7.40–7.54 (m, 16H, *d, f, g, o, p*), 7.63–7.65(d, *J* = 8.0 Hz, 2H, *r*), 7.69–7.70 (d, *J* = 7.5 Hz, 4H, *q*), 8.49 (s, 2H, *n*), 8.63–8.65 (d, *J* = 8.0 Hz, 2H, *s*), 8.80–8.81 (d, *J* = 6.3 Hz, 2H, *b*), 10.23 (s, 1H, *a*); ^{13}C NMR peaks were barely detected because of the law solubility of the title compound.; ^{11}B NMR (128 MHz, $CDCl_3$) δ 40.8; HRMS (MALDI-TOF/MS) *m/z* $[M]^+$ calcd for $C_{90}H_{59}B_3N_6$ 1256.511; observed 1256.510.

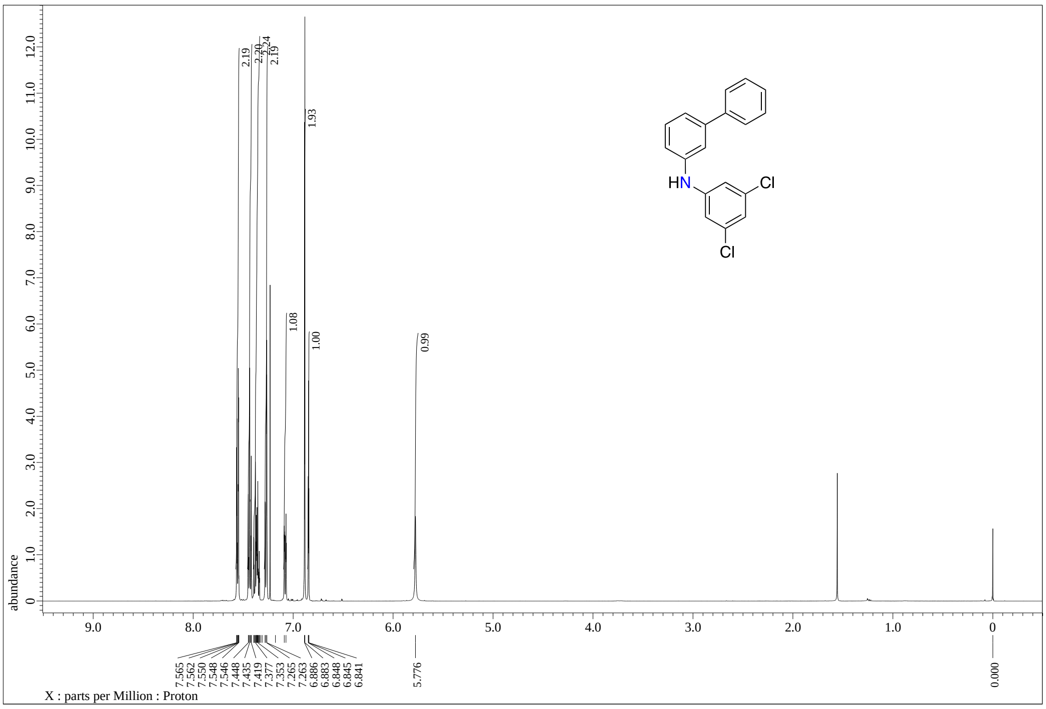

Supplementary Figure 2. ^{1}H NMR spectrum of **5** in $CDCl_3$ at 25 ºC.

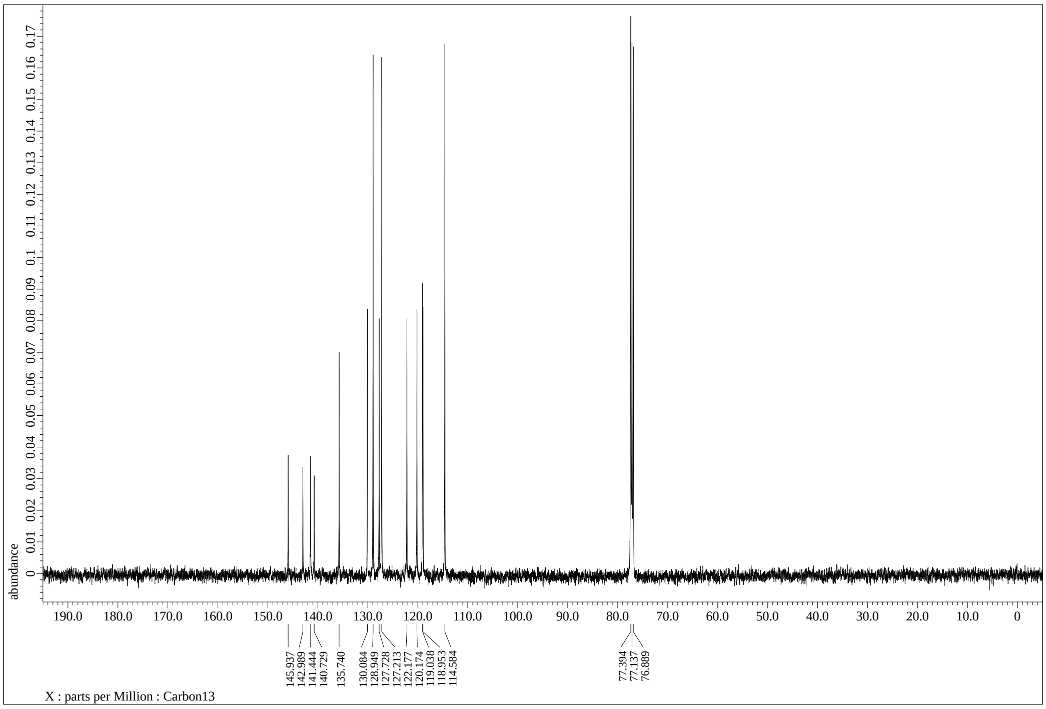

Supplementary Figure 3. ^{13}C NMR spectrum of **5** in $CDCl_3$ at 25 ºC.

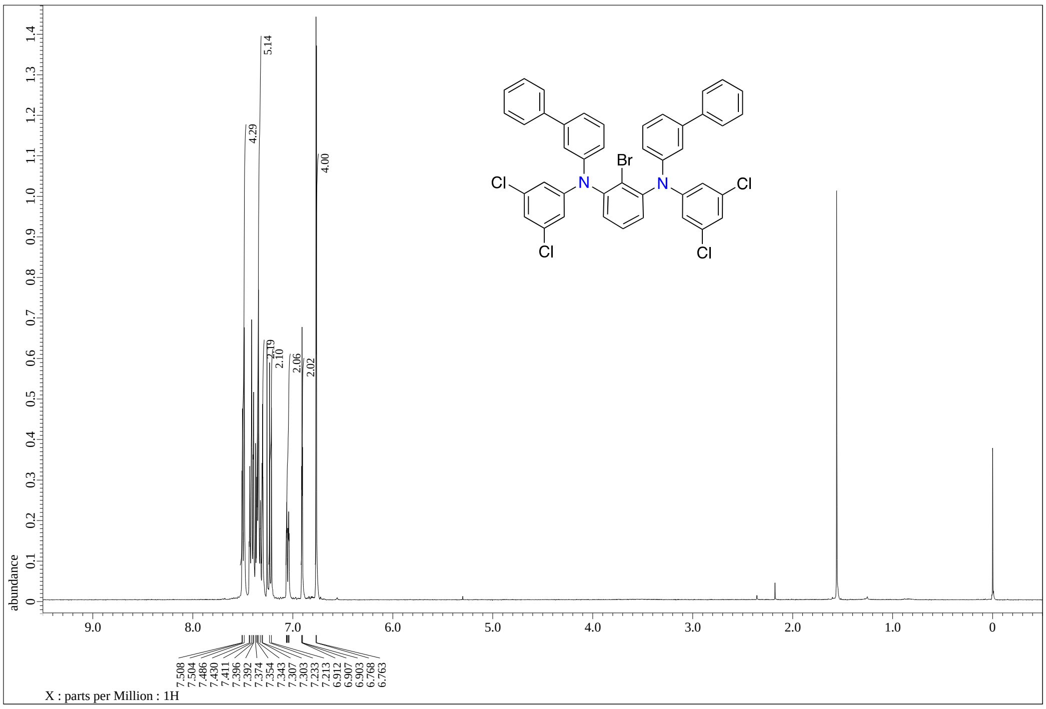

Supplementary Figure 4. ^{1}H NMR spectrum of **6** in $CDCl_3$ at 25 ºC.

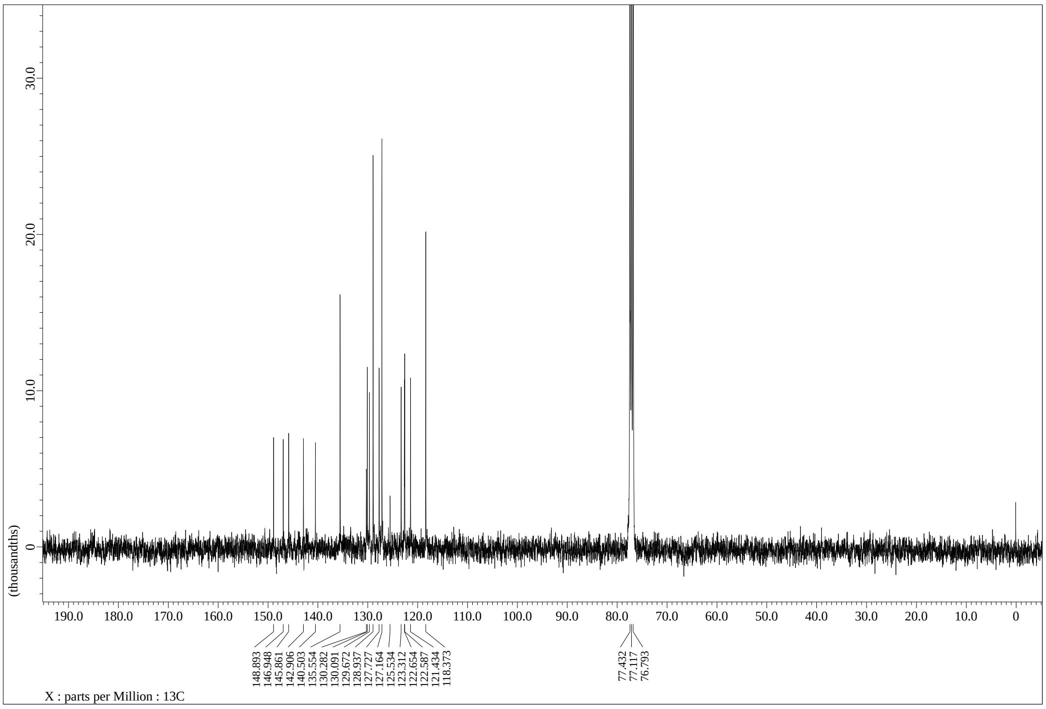

Supplementary Figure 5. ^{13}C NMR spectrum of **6** in $CDCl_3$ at 25 ºC.

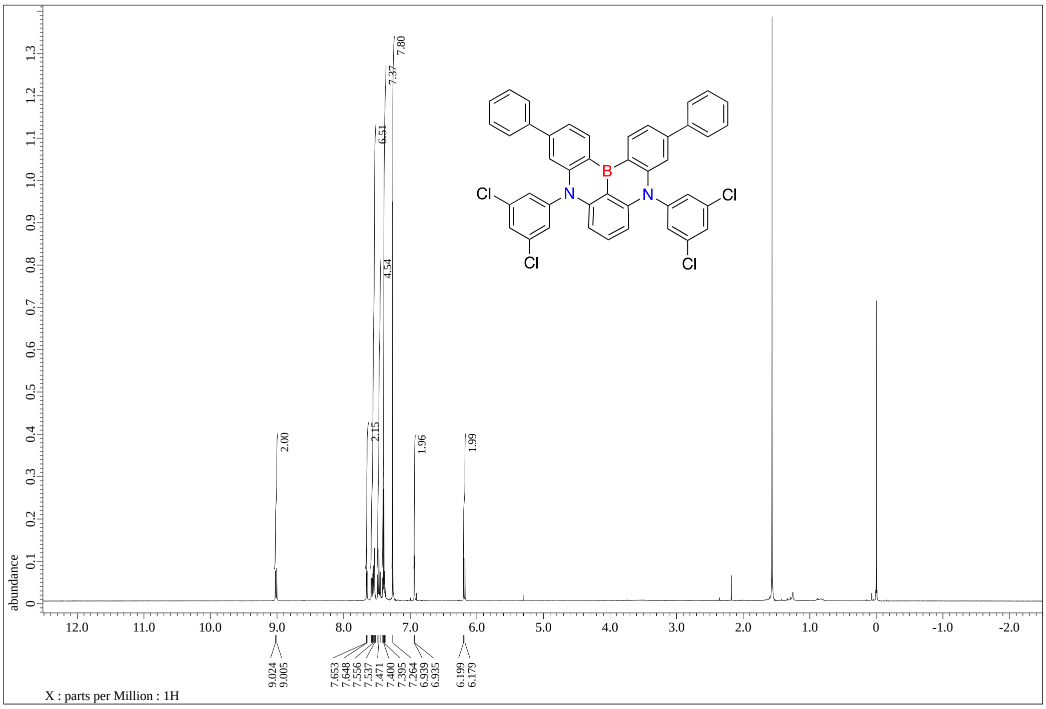

Supplementary Figure 6. ^{1}H NMR spectrum of **7** in $CDCl_3$ at 25 ºC.

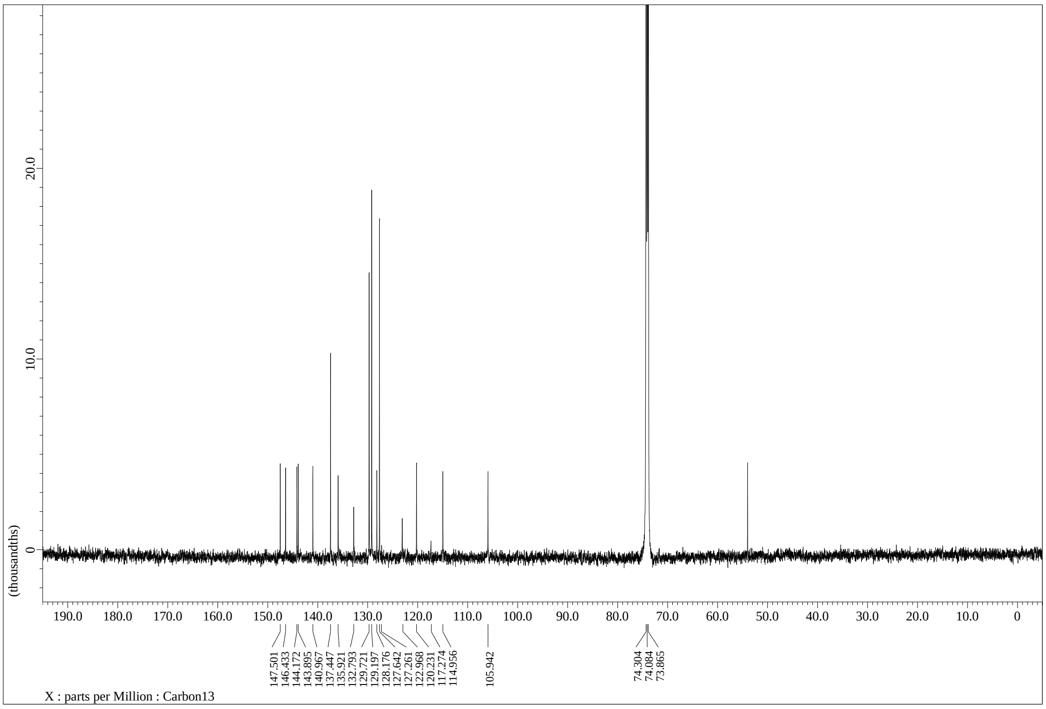

Supplementary Figure 7. ^{13}C NMR spectrum of **7** in CD_2Cl_4 at 120 ºC.

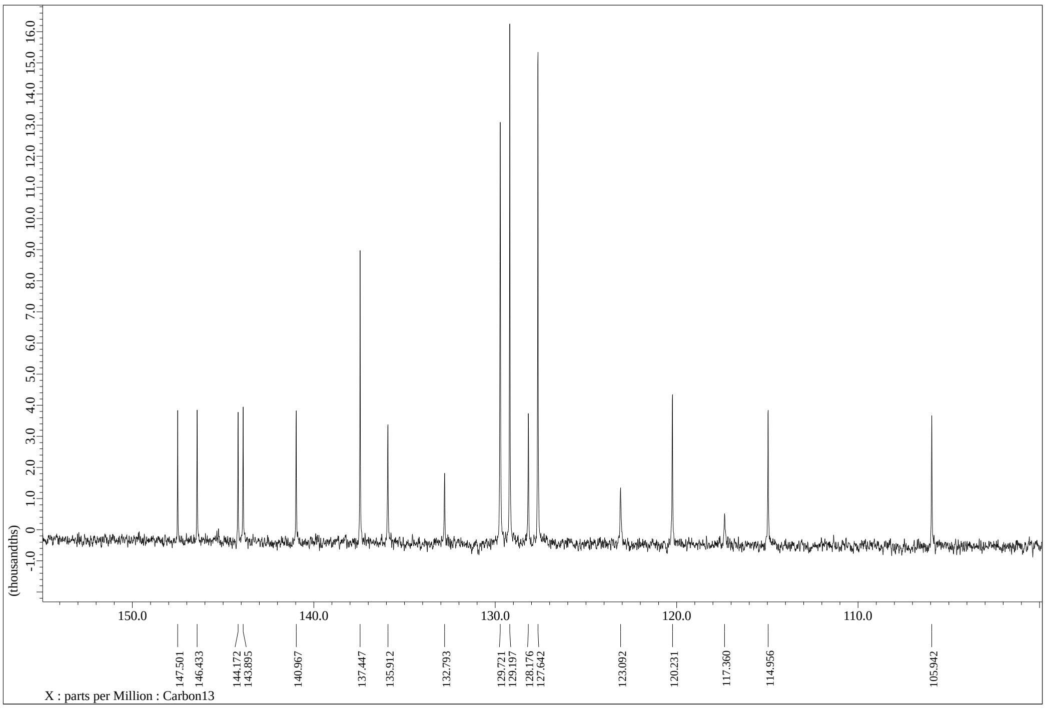

Supplementary Figure 8. Enlarged ^{13}C NMR spectrum of **7** in CD_2Cl_4 at 120 ºC.

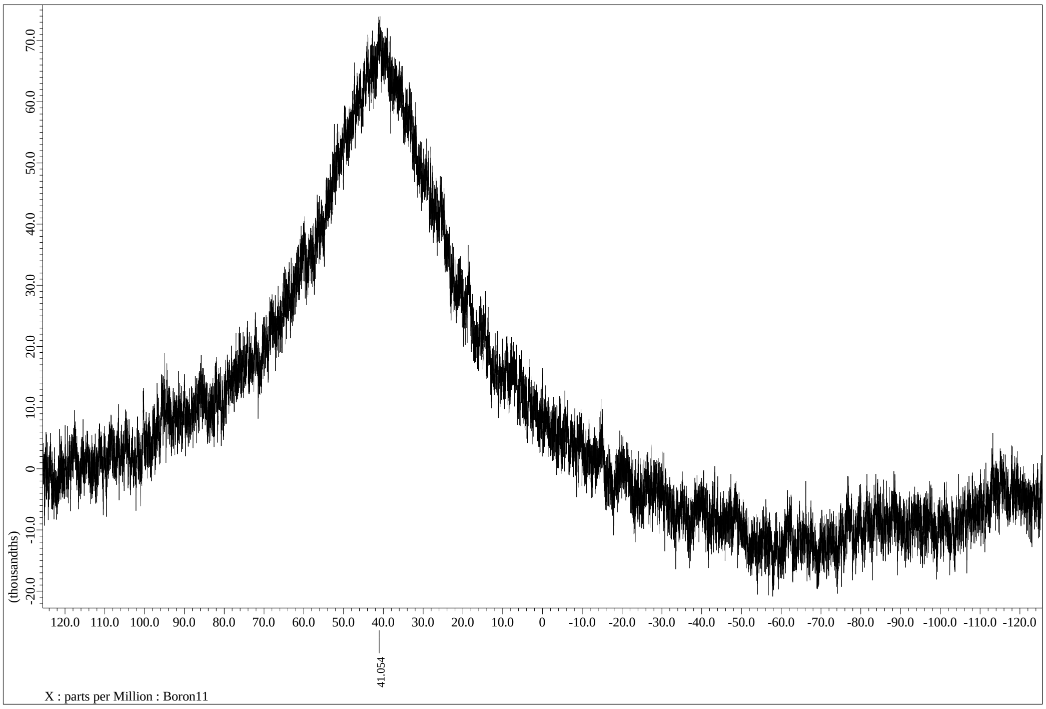

Supplementary Figure 9. ^{11}B NMR spectrum of **7** in CD_2Cl_4 at 120 ºC.

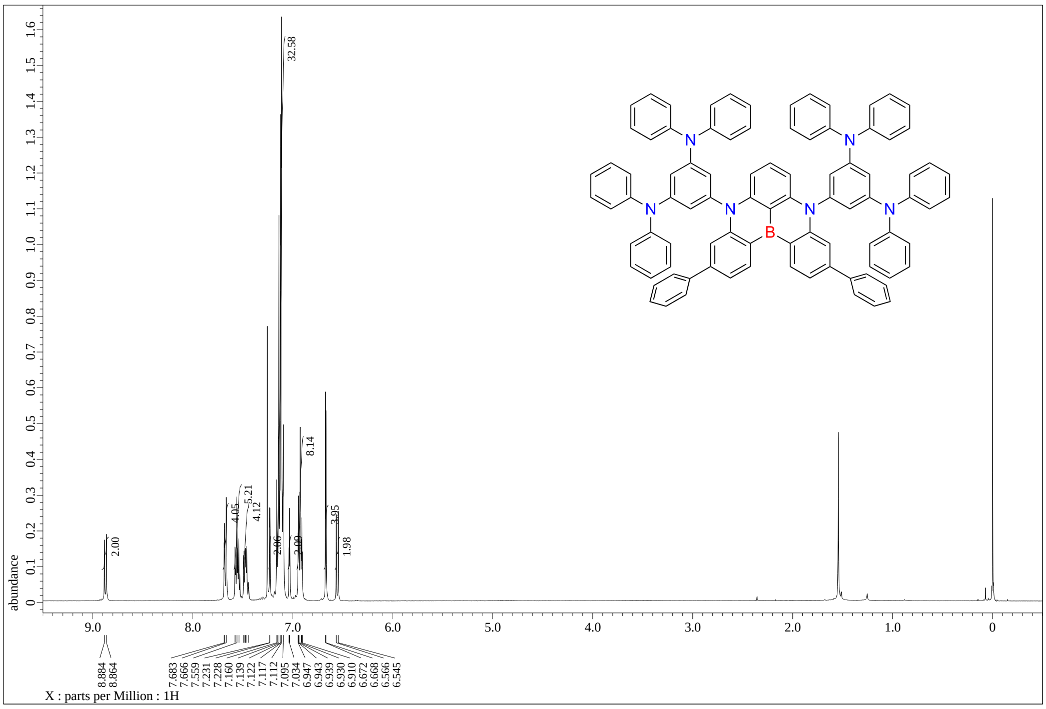

Supplementary Figure 10. ^{1}H NMR spectrum of **8** in $CDCl_3$ at 25 ºC.

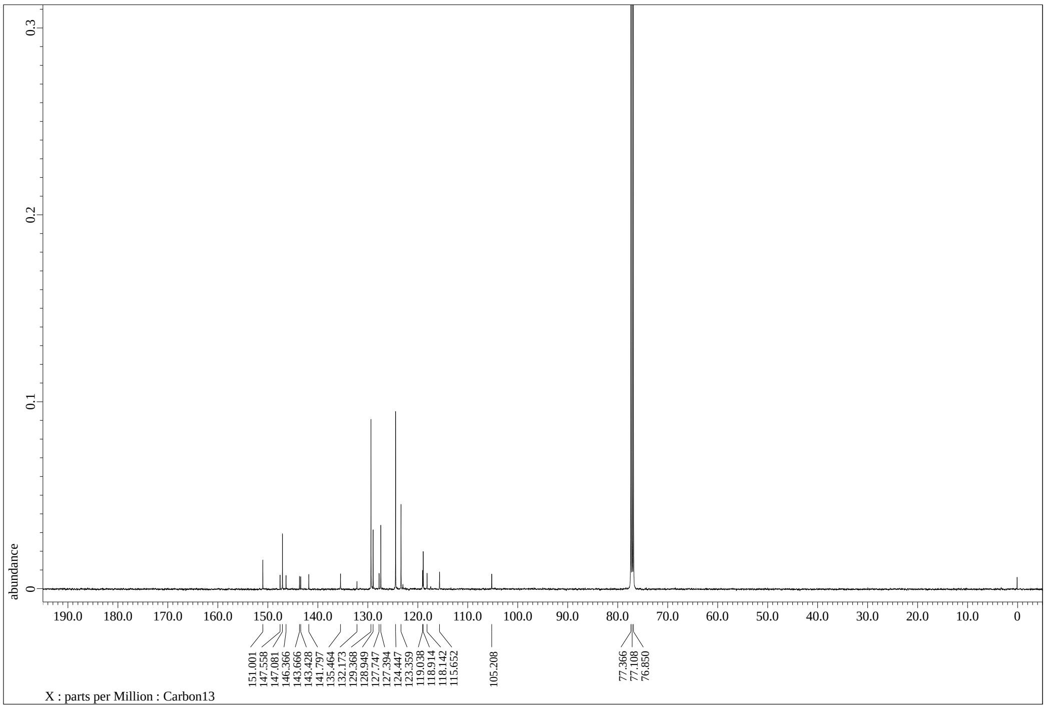

Supplementary Figure 11. ^{13}C NMR spectrum of **8** in $CDCl_3$ at 25 ºC.

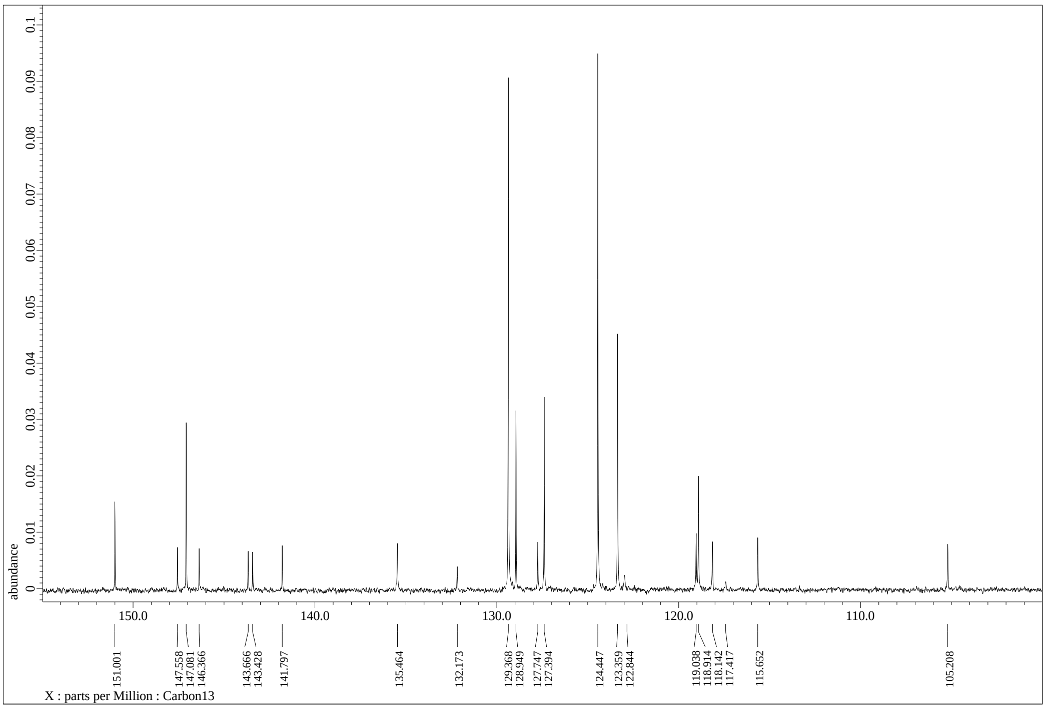

Supplementary Figure 12. Enlarged ^{13}C NMR spectrum of **8** in $CDCl_3$ at 25 ºC.

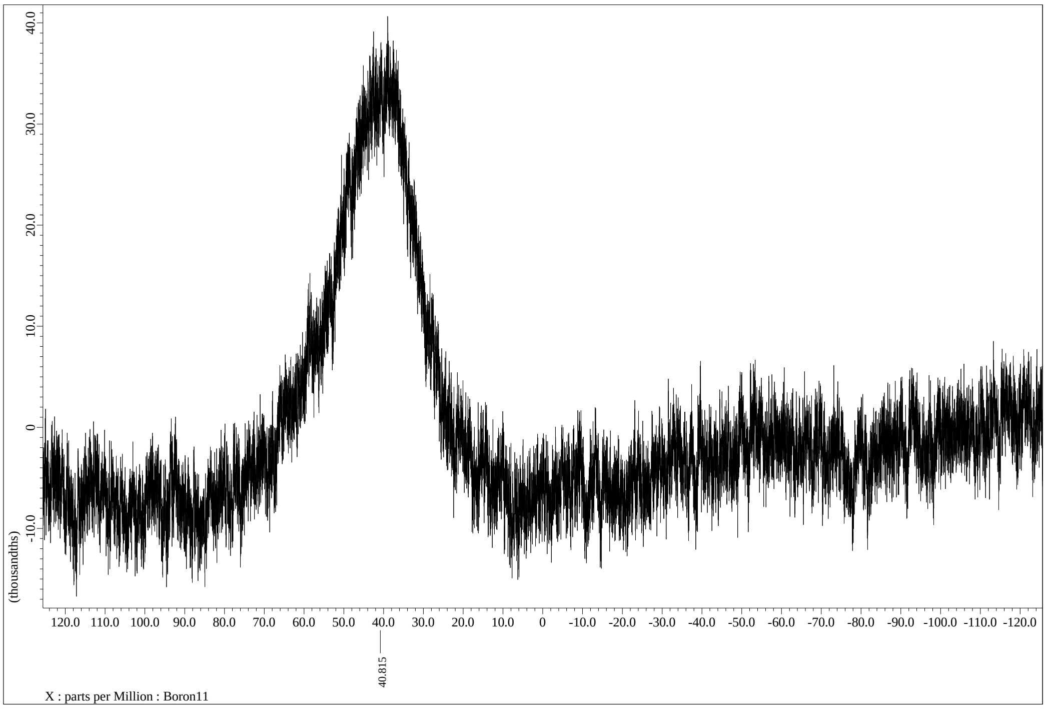

Supplementary Figure 13. ^{11}B NMR spectrum of **8** in $CDCl_3$ at 25 ºC.

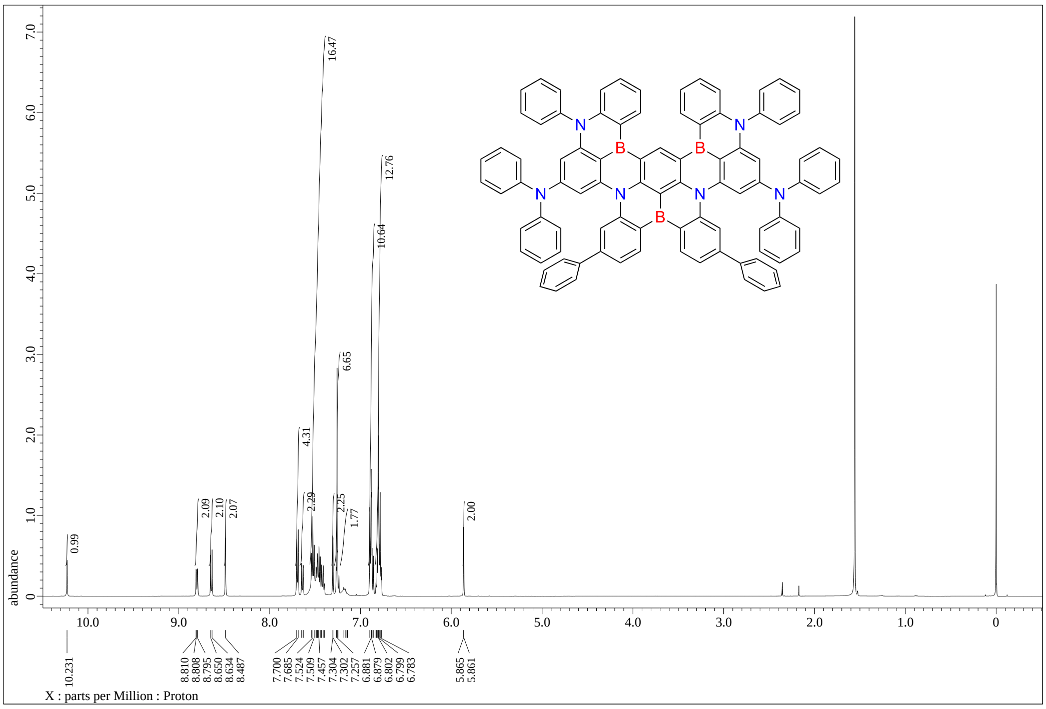

Supplementary Figure 14. ^{1}H NMR spectrum of **ω-DABNA-PH** in $CDCl_3$ at 25 ºC.

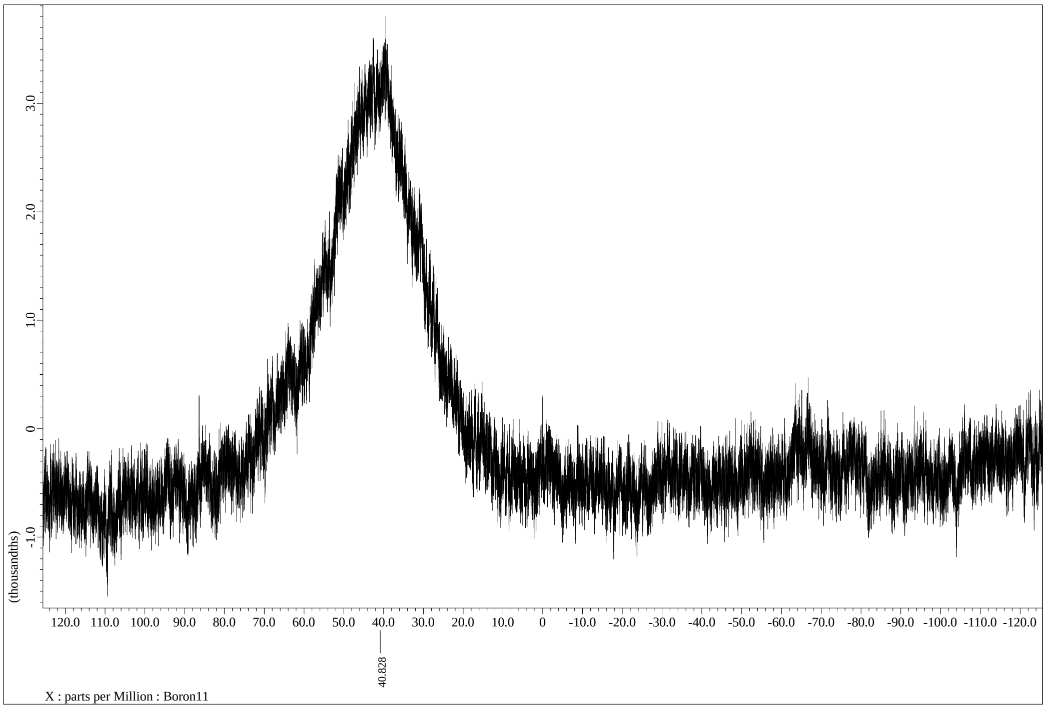

Supplementary Figure 15. ^{11}B NMR spectrum of **ω-DABNA-PH** in $CDCl_3$ at 25 ºC.

Supplementary Note 2

All calculations were performed with Gaussian 16 (Revision B.01), and PySCF packages unless otherwise noted. The DFT method was employed using the M062X functional. Structures were optimized with the 6-31G(d) basis set. The time-dependent density functional theory (TD-DFT) calculation was conducted at the M062X/6-31G(d) level after the geometry optimization at the M062X/6-31G(d) level of theory. Grimme's B2PLYP-type double-hybrid exchange-correlation functional has the following energy form: $E_{xc}(c_x, c_c) = (1 - c_x)E_x^{\mathrm{B88}} + c_x E_x^{\mathrm{HF}} + (1 - c_c)E_c^{\mathrm{LYP}} + c_c E_c^{\mathrm{PT2}}$, where E_x^{B88} and E_c^{LYP} are the Becke88 exchange and Lee–Yang–Parr correlation energies, respectively; E_x^{HF} is the Hartree–Fock (HF) exchange energy; E_c^{PT2} is the second-order perturbation correlation energy; and coefficients c_x and c_x are exchange and correlation mixing parameters. We here denote B2LYP/B2PLYP variants by B2LYP/B2PLYP (c_x = 0.40, c_c = 0.23). Virtual MOs with energies larger than 0.6 Ha were omitted for DH calculations. Transition energies and ΔE_{ST} values were estimated for optimized S_0 geometries within double-hybrid time-dependent density functional theory (DH-TDDFT), at the TDA-B2PLYP (c_x = 0.40, c_c = 0.23)/cc-pVDZ//M062X/6-31G(d) level of theory. Due to the limit of our computation resource, we omitted virtual orbitals with energies higher than 0.6 Hartree and computed two-electron integrals in the resolution-of-the-identity approximation for CIS(D) calculations. All the DH-TDDFT computations were conducted using PySCF with in-house CIS(D) submodules.[20] SOC matrix elements between singlet and triplet states were evaluated in the framework of the effective one-electron formalism of Gao and coworkers[21], in which we adopted CI (configuration interaction) amplitudes at the TDA-B2LYP(c_x = 0.40, c_c = 0.23)/cc-pVDZ level in combination with effective nuclear charges developed by Koseki and coworkers[22]. We employed the Libcint library[23], which is part of PySCF, for the calculation of one-electron integrals.

Supplementary Table 2. Summary of TD-DFT calculation for ω-DABNA analogs at the M062X/6-31G(d) level of theory (Gaussian 16)

compound	HOMO–1 [eV]	HOMO [eV]	LUMO [eV]	LUMO+1 [eV]	$\lambda_{(S0\text{-}S1)}$ [nm]	E_S[a] [eV]	f[b]	$\lambda_{(S0\text{-}T1)}$ [nm]	E_T[c] [eV]	ΔE_{ST}[d] [eV]
**ω-DABNA**	–6.14	–5.74	–0.81	–0.31	395	3.14	0.8595	444	2.79	0.35
**ω-DABNA-M**	–5.98	–5.60	–0.70	–0.20	397	3.12	0.8402	411	2.78	0.34
**ω-DABNA-PH**	–6.25	–5.86	–1.05	–0.46	403	3.08	0.8161	454	2.73	0.35

[a]Singlet–singlet excitation energy. [b]Oscillator strength. [c]Singlet–triplet excitation energy. [d]Energy gap between S_1 and T_1 states.

Supplementary Table 3. Summary of calculated transitions for ω-DABNA analogs.

compound	$S_1/T_1/T_2$ energy	coefficient of orbital					
**ω-DABNA**	S_1: 3.14 eV (395 nm)	HOMO–3 → LUMO+1 (–0.12810)	HOMO–1 → LUMO+1 (0.11072)	HOMO → LUMO (0.66157)			
	T_1: 2.79 eV (444 nm)	HOMO–3 → LUMO+1 (–0.12503)	HOMO–1 → LUMO+1 (0.10670)	HOMO → LUMO (0.64738)			
	T_2: 3.04 eV (407 nm)	HOMO–4 → LUMO (0.13401)	HOMO–3 → LUMO (–0.25102)	HOMO–3 → LUMO+2 (0.15981)	HOMO–1 → LUMO (0.18011)	HOMO–1 → LUMO+2 (–0.13753)	HOMO → LUMO+1 (0.53832)
**ω-DABNA-M**	S_1: 3.12 eV (397 nm)	HOMO–3 → LUMO+1 (–0.12348)	HOMO–1 → LUMO+1 (0.10812)	HOMO → LUMO (0.66045)			
	T_1: 2.78 eV (446 nm)	HOMO–3 → LUMO+1 (–0.13327)	HOMO–1 → LUMO+1 (0.11145)	HOMO → LUMO (0.64318)			
	T_2: 3.01 eV (412 nm)	HOMO–4 → LUMO (0.11588)	HOMO–3 → LUMO (–0.25795)	HOMO–3 → LUMO+2 (0.17156)	HOMO–1 → LUMO (0.18011)	HOMO–1 → LUMO+2 (–0.14781)	HOMO → LUMO+1 (0.52707)
**ω-DABNA-PH**	S_1: 3.08 eV (403 nm)	HOMO–3 → LUMO+1 (–0.14790)	HOMO → LUMO (0.65615)				
	T_1: 2.73 eV (454 nm)	HOMO–3 → LUMO+1 (–0.14311)	HOMO → LUMO (0.63540)	HOMO → LUMO+2 (–0.10905)			
	T_2: 3.00 eV (414 nm)	HOMO–4 → LUMO (0.10231)	HOMO–3 → LUMO (–0.29115)	HOMO–3 → LUMO+2 (0.21035)	HOMO–1 → LUMO (0.15356)	HOMO–1 → LUMO+2 (–0.11472)	HOMO → LUMO+1 (0.50832)

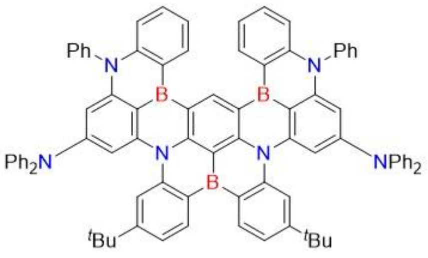

ω-DABNA

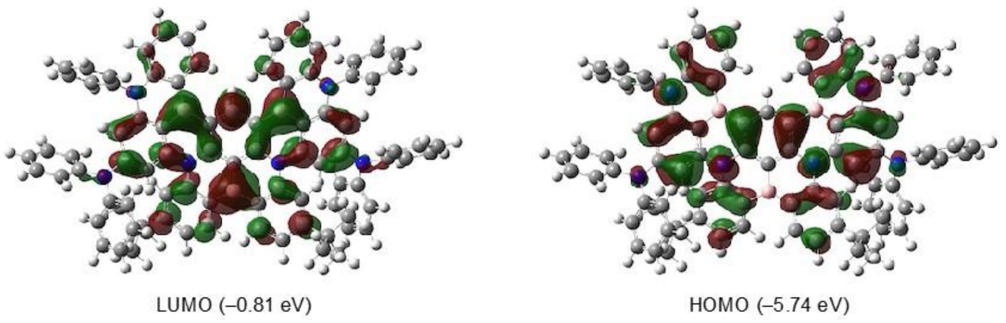

Supplementary Figure 16. Kohn–Sham frontier orbitals of **ω-DABNA** calculated at the M062X/6-31G(d) level of theory (Gaussian 16).

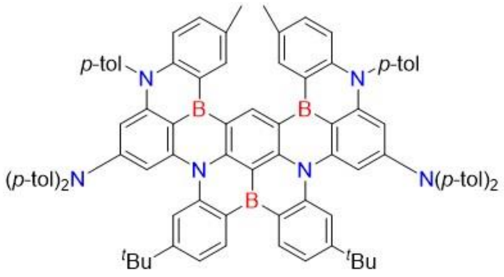

ω-DABNA-M

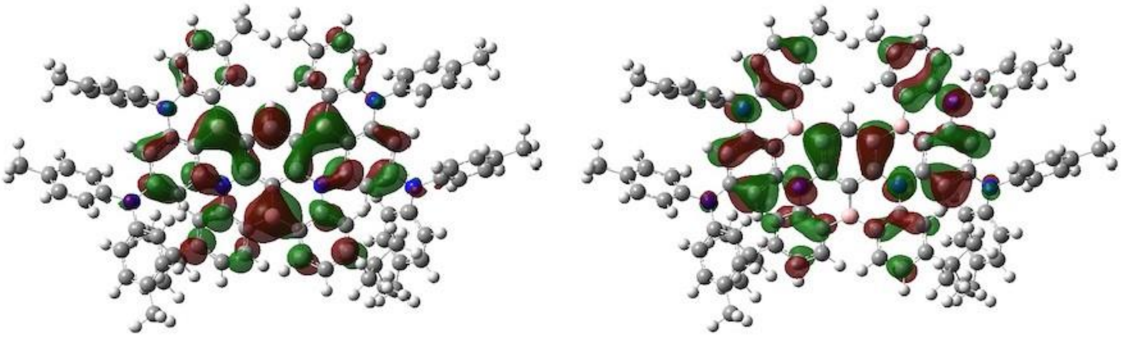

LUMO (–0.70 eV) HOMO (–5.60 eV)

Supplementary Figure 17. Kohn–Sham frontier orbitals of **ω-DABNA-M** calculated at the M062X/6-31G(d) level of theory (Gaussian 16).

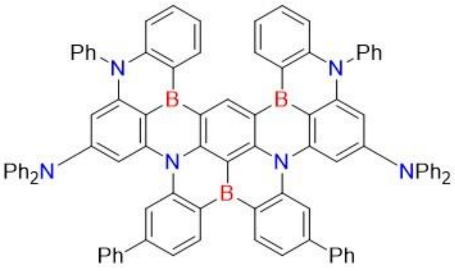

ω-DABNA-PH

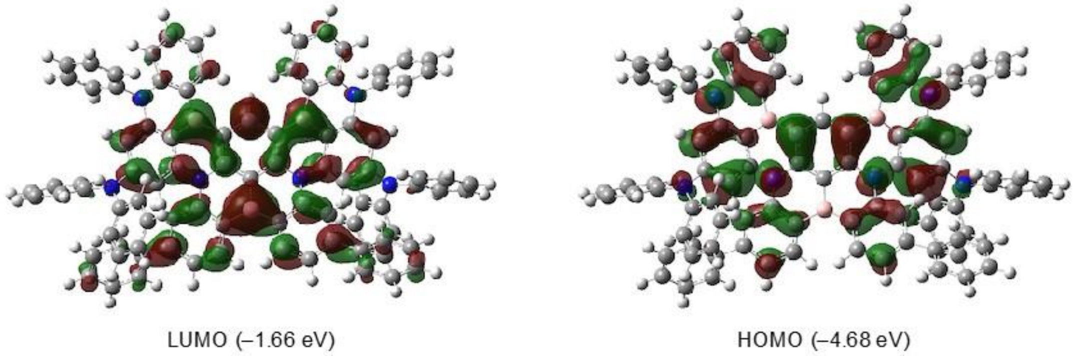

LUMO (–1.66 eV) HOMO (–4.68 eV)

Supplementary Figure 18. Kohn–Sham frontier orbitals of **ω-DABNA-PH** calculated at the B3LYP/6-31G(d) level of theory (Gaussian 16).

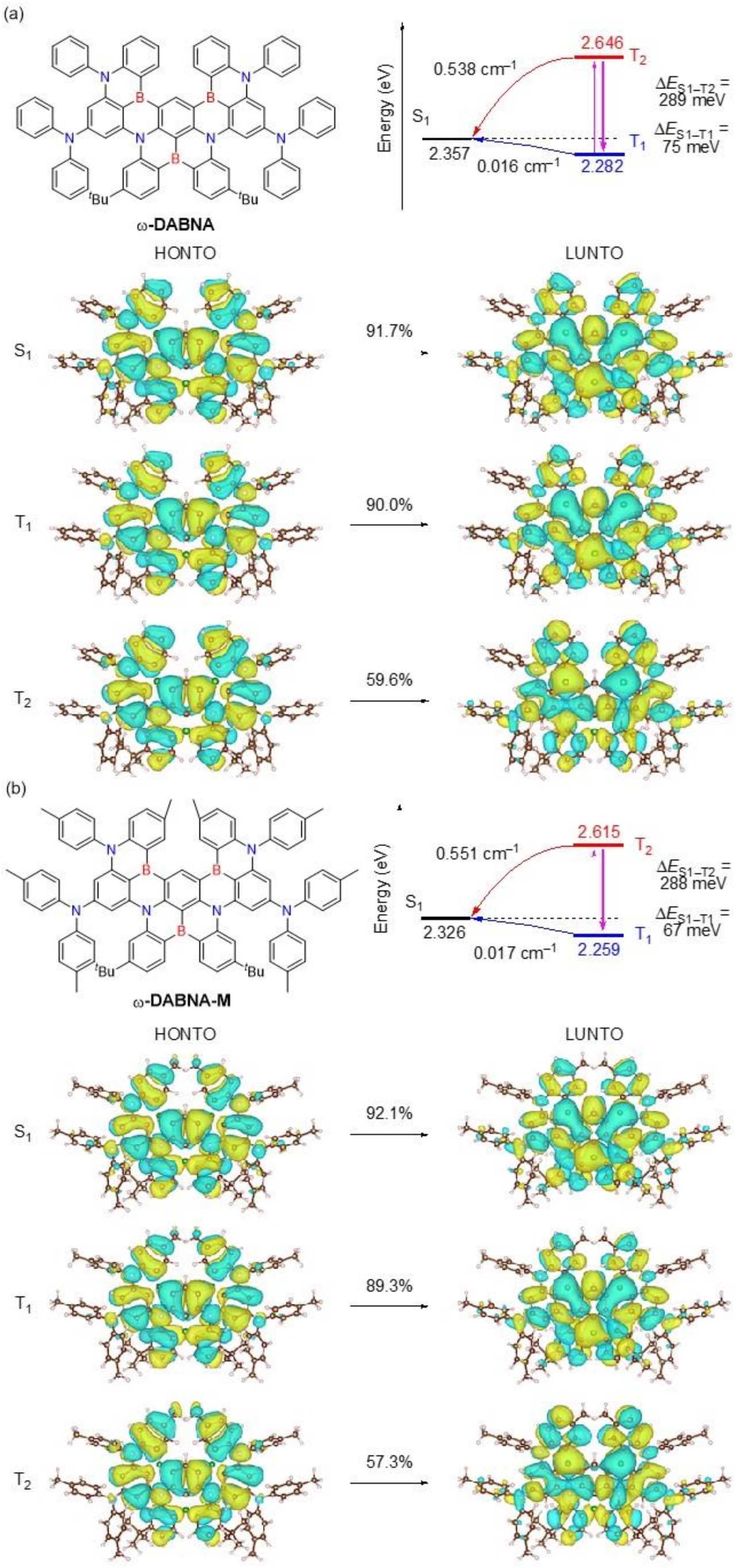
(a)
N
B
tBu
ω-DABNA
Energy (eV)
0.538 cm−1
2.646
T2
S1
T1
2.357
0.016 cm−1
2.282
ΔES1–T2 = 289 meV
ΔES1–T1 = 75 meV
HONTO
LUNTO
S1
91.7%
T1
90.0%
T2
59.6%
(b)
ω-DABNA-M
Energy (eV)
0.551 cm−1
2.615
T2
S1
T1
2.326
0.017 cm−1
2.259
ΔES1–T2 = 288 meV
ΔES1–T1 = 67 meV
HONTO
LUNTO
S1
92.1%
T1
89.3%
T2
57.3%

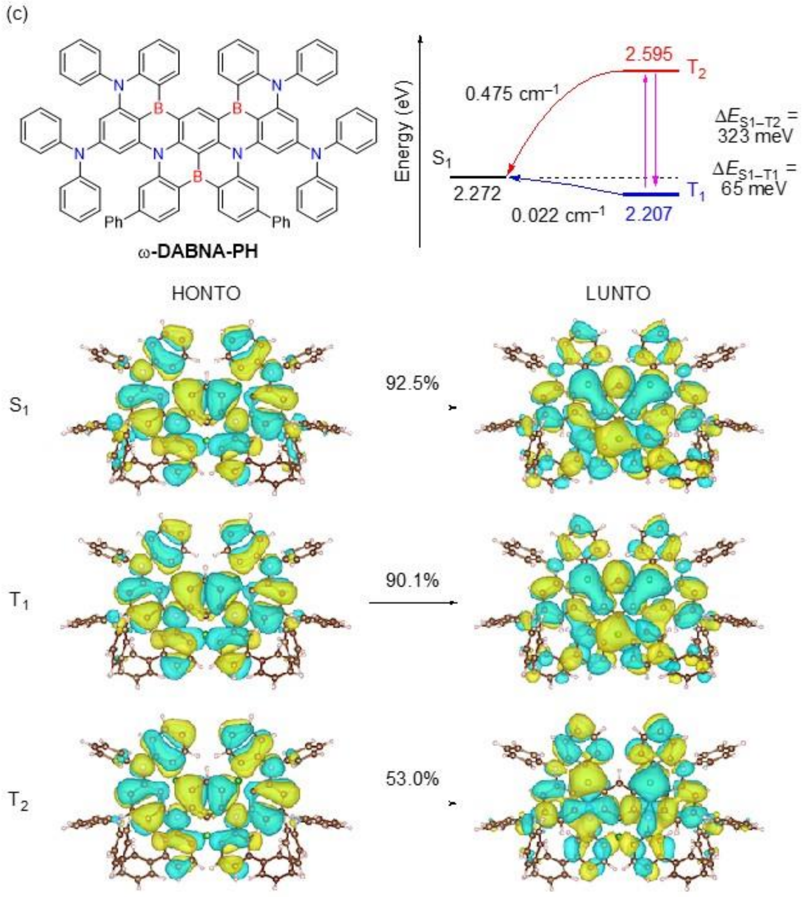

Supplementary Figure 19. Molecular structure, energy level diagram, and associated natural transition orbitals (NTOs) for singlet and triplet excited states. (a) **ω-DABNA**, (b) **ω-DABNA-M**, and (c) **ω-DABNA-PH** at the S_0 geometry. Transition energies for S_1, T_1, and T_2, NTOs, and SOC matrix elements between the T_n and S_n states were calculated at the TDA-B2PLYP(cx = 0.40, cc = 0.23)/cc-pVDZ//M062X/6-31G(d) level of theory.

Supplementary Table 4. Cartesian Coordinate of ω-DABNA (S_0, C_2 symmetry).
E(M062X/6-31G(d)) = –3719.289731 hartree

Center Number	Atomic Number	Atomic Type	Coordinates (Angstroms) X	Y	Z
1	5	0	0.000000	0.000000	-2.155731
2	7	0	-0.412687	-2.389376	-0.645740
3	7	0	0.412687	2.389376	-0.645740
4	6	0	0.223659	1.199648	0.076166
5	6	0	0.000000	0.000000	-0.640433

6	6	0	-0.223659	-1.199648	0.076166
7	6	0	-0.257411	-1.215677	1.493858
8	6	0	0.000000	0.000000	2.133670
9	6	0	0.257411	1.215677	1.493858
10	1	0	0.000000	0.000000	3.218355
11	6	0	0.879019	2.353795	-1.989244
12	6	0	1.641195	3.431445	-2.476039
13	6	0	0.640962	1.235204	-2.806921
14	6	0	2.086428	3.477042	-3.787589
15	1	0	1.877474	4.243544	-1.803544
16	6	0	1.146641	1.276044	-4.121821
17	6	0	1.825422	2.370179	-4.617316
18	1	0	2.178099	2.364256	-5.644927
19	6	0	-0.879019	-2.353795	-1.989244
20	6	0	-0.640962	-1.235204	-2.806921
21	6	0	-1.641195	-3.431445	-2.476039
22	6	0	-1.146641	-1.276044	-4.121821
23	6	0	-2.086428	-3.477042	-3.787589
24	1	0	-1.877474	-4.243544	-1.803544
25	6	0	-1.825422	-2.370179	-4.617316
26	1	0	-2.178099	-2.364256	-5.644927
27	6	0	0.114347	3.640911	-0.040578
28	6	0	0.231877	3.769287	1.359322
29	6	0	-0.295613	4.716665	-0.823303
30	6	0	0.000000	5.035638	1.943591
31	6	0	-0.528594	5.959320	-0.220830
32	1	0	-0.419173	4.614301	-1.892820
33	6	0	-0.393898	6.124492	1.158571
34	1	0	-0.597909	7.092304	1.595720
35	6	0	-0.114347	-3.640911	-0.040578
36	6	0	-0.231877	-3.769287	1.359322
37	6	0	0.295613	-4.716665	-0.823303
38	6	0	0.000000	-5.035638	1.943591
39	6	0	0.528594	-5.959320	-0.220830
40	1	0	0.419173	-4.614301	-1.892820
41	6	0	0.393898	-6.124492	1.158571
42	1	0	0.597909	-7.092304	1.595720
43	1	0	1.012587	0.402793	-4.754189
44	1	0	-1.012587	-0.402793	-4.754189
45	7	0	-0.886874	7.065584	-1.017973
46	7	0	0.886874	-7.065584	-1.017973
47	6	0	-0.461488	8.374951	-0.675418
48	6	0	0.819777	8.607259	-0.162159
49	6	0	-1.325273	9.456730	-0.874209

50	6	0	1.216365	9.899264	0.163759
51	1	0	1.494591	7.769352	-0.016706
52	6	0	-0.912403	10.747501	-0.564103
53	1	0	-2.317667	9.273278	-1.274564
54	6	0	0.356488	10.976667	-0.037452
55	1	0	2.212625	10.064593	0.563128
56	1	0	-1.593956	11.577349	-0.724202
57	1	0	0.672760	11.984391	0.211546
58	6	0	0.461488	-8.374951	-0.675418
59	6	0	-0.819777	-8.607259	-0.162159
60	6	0	1.325273	-9.456730	-0.874209
61	6	0	-1.216365	-9.899264	0.163759
62	1	0	-1.494591	-7.769352	-0.016706
63	6	0	0.912403	-10.747501	-0.564103
64	1	0	2.317667	-9.273278	-1.274564
65	6	0	-0.356488	-10.976667	-0.037452
66	1	0	-2.212625	-10.064593	0.563128
67	1	0	1.593956	-11.577349	-0.724202
68	1	0	-0.672760	-11.984391	0.211546
69	6	0	-1.535138	6.869775	-2.266143
70	6	0	-2.666135	6.052278	-2.349953
71	6	0	-1.046034	7.489978	-3.419892
72	6	0	-3.288526	5.848469	-3.576230
73	1	0	-3.040648	5.574652	-1.449627
74	6	0	-1.686228	7.295269	-4.639844
75	1	0	-0.167666	8.124973	-3.349560
76	6	0	-2.805495	6.470696	-4.725566
77	1	0	-4.162699	5.207124	-3.630361
78	1	0	-1.297709	7.781918	-5.529404
79	1	0	-3.297514	6.314129	-5.679934
80	6	0	1.535138	-6.869775	-2.266143
81	6	0	2.666135	-6.052278	-2.349953
82	6	0	1.046034	-7.489978	-3.419892
83	6	0	3.288526	-5.848469	-3.576230
84	1	0	3.040648	-5.574652	-1.449627
85	6	0	1.686228	-7.295269	-4.639844
86	1	0	0.167666	-8.124973	-3.349560
87	6	0	2.805495	-6.470696	-4.725566
88	1	0	4.162699	-5.207124	-3.630361
89	1	0	1.297709	-7.781918	-5.529404
90	1	0	3.297514	-6.314129	-5.679934
91	6	0	2.831999	4.691884	-4.343848
92	6	0	2.016870	5.272735	-5.512942
93	6	0	3.008606	5.795901	-3.295317

94	6	0	4.220568	4.264551	-4.845898
95	1	0	1.884250	4.540716	-6.315434
96	1	0	1.024145	5.587323	-5.172376
97	1	0	2.531476	6.144636	-5.932944
98	1	0	3.611464	5.455874	-2.446474
99	1	0	3.521863	6.650398	-3.748329
100	1	0	2.041756	6.147526	-2.914796
101	1	0	4.756434	5.128032	-5.255899
102	1	0	4.816204	3.845772	-4.028307
103	1	0	4.149366	3.508300	-5.633538
104	6	0	-2.831999	-4.691884	-4.343848
105	6	0	-2.016870	-5.272735	-5.512942
106	6	0	-3.008606	-5.795901	-3.295317
107	6	0	-4.220568	-4.264551	-4.845898
108	1	0	-1.884250	-4.540716	-6.315434
109	1	0	-1.024145	-5.587323	-5.172376
110	1	0	-2.531476	-6.144636	-5.932944
111	1	0	-3.611464	-5.455874	-2.446474
112	1	0	-3.521863	-6.650398	-3.748329
113	1	0	-2.041756	-6.147526	-2.914796
114	1	0	-4.756434	-5.128032	-5.255899
115	1	0	-4.816204	-3.845772	-4.028307
116	1	0	-4.149366	-3.508300	-5.633538
117	7	0	0.146531	5.184466	3.324263
118	7	0	-0.146531	-5.184466	3.324263
119	6	0	0.596285	4.165876	4.179829
120	6	0	0.864465	4.475679	5.529015
121	6	0	0.788062	2.843854	3.706135
122	6	0	1.341405	3.499681	6.385191
123	1	0	0.713529	5.481397	5.901566
124	6	0	1.303064	1.895168	4.611173
125	6	0	1.579059	2.199131	5.931828
126	1	0	1.547061	3.762383	7.418902
127	1	0	1.515134	0.895376	4.244709
128	1	0	1.977849	1.445118	6.602309
129	6	0	-0.596285	-4.165876	4.179829
130	6	0	-0.864465	-4.475679	5.529015
131	6	0	-0.788062	-2.843854	3.706135
132	6	0	-1.341405	-3.499681	6.385191
133	1	0	-0.713529	-5.481397	5.901566
134	6	0	-1.303064	-1.895168	4.611173
135	6	0	-1.579059	-2.199131	5.931828
136	1	0	-1.547061	-3.762383	7.418902
137	1	0	-1.515134	-0.895376	4.244709

138	1	0	-1.977849	-1.445118	6.602309
139	5	0	0.474739	2.553673	2.224817
140	5	0	-0.474739	-2.553673	2.224817
141	6	0	-0.098009	6.484741	3.877988
142	6	0	-1.330222	6.766486	4.460819
143	6	0	0.892061	7.462619	3.808774
144	6	0	-1.571258	8.035238	4.981620
145	1	0	-2.085104	5.987069	4.499521
146	6	0	0.643531	8.732144	4.321829
147	1	0	1.842307	7.217770	3.343106
148	6	0	-0.586910	9.018262	4.910033
149	1	0	-2.530897	8.257236	5.437510
150	1	0	1.410033	9.498180	4.260936
151	1	0	-0.779353	10.008661	5.310183
152	6	0	0.098009	-6.484741	3.877988
153	6	0	1.330222	-6.766486	4.460819
154	6	0	-0.892061	-7.462619	3.808774
155	6	0	1.571258	-8.035238	4.981620
156	1	0	2.085104	-5.987069	4.499521
157	6	0	-0.643531	-8.732144	4.321829
158	1	0	-1.842307	-7.217770	3.343106
159	6	0	0.586910	-9.018262	4.910033
160	1	0	2.530897	-8.257236	5.437510
161	1	0	-1.410033	-9.498180	4.260936
162	1	0	0.779353	-10.008661	5.310183

Supplementary Table 5. Cartesian Coordinate of ω-DABNA-M (S_0, C_2 symmetry).
E(M062X/6-31G(d)) = –4033.664706 hartree

Center	Atomic	Atomic	Coordinates (Angstroms)		
Number	Number	Type	X	Y	Z
1	5	0	0.000000	0.000000	-2.295863
2	7	0	-0.522686	-2.371321	-0.788316
3	7	0	0.522686	2.371321	-0.788316
4	6	0	0.269339	1.192500	-0.067453
5	6	0	0.000000	0.000000	-0.782133
6	6	0	-0.269339	-1.192500	-0.067453
7	6	0	-0.279518	-1.211711	1.348966
8	6	0	0.000000	0.000000	1.982652
9	6	0	0.279518	1.211711	1.348966

10	1	0	0.000000	0.000000	3.063131
11	6	0	1.002913	2.304500	-2.126029
12	6	0	1.835449	3.331851	-2.606006
13	6	0	0.707218	1.200001	-2.944761
14	6	0	2.296826	3.346929	-3.913021
15	1	0	2.112947	4.129442	-1.931566
16	6	0	1.225968	1.207804	-4.254835
17	6	0	1.975634	2.257509	-4.744563
18	1	0	2.337687	2.228395	-5.768574
19	6	0	-1.002913	-2.304500	-2.126029
20	6	0	-0.707218	-1.200001	-2.944761
21	6	0	-1.835449	-3.331851	-2.606006
22	6	0	-1.225968	-1.207804	-4.254835
23	6	0	-2.296826	-3.346929	-3.913021
24	1	0	-2.112947	-4.129442	-1.931566
25	6	0	-1.975634	-2.257509	-4.744563
26	1	0	-2.337687	-2.228395	-5.768574
27	6	0	0.265112	3.638267	-0.193249
28	6	0	0.323294	3.761720	1.213250
29	6	0	-0.057647	4.731495	-0.993105
30	6	0	0.105031	5.036815	1.783866
31	6	0	-0.270247	5.985592	-0.403064
32	1	0	-0.135758	4.632005	-2.066423
33	6	0	-0.204595	6.139919	0.982475
34	1	0	-0.401066	7.109910	1.415770
35	6	0	-0.265112	-3.638267	-0.193249
36	6	0	-0.323294	-3.761720	1.213250
37	6	0	0.057647	-4.731495	-0.993105
38	6	0	-0.105031	-5.036815	1.783866
39	6	0	0.270247	-5.985592	-0.403064
40	1	0	0.135758	-4.632005	-2.066423
41	6	0	0.204595	-6.139919	0.982475
42	1	0	0.401066	-7.109910	1.415770
43	1	0	1.044038	0.343048	-4.887245
44	1	0	-1.044038	-0.343048	-4.887245
45	7	0	-0.539044	7.105555	-1.210654
46	7	0	0.539044	-7.105555	-1.210654
47	6	0	-0.226313	8.417461	-0.764536
48	6	0	0.992145	8.695882	-0.134352
49	6	0	-1.136266	9.457117	-0.963296
50	6	0	1.269675	9.980701	0.311218
51	1	0	1.707181	7.893216	0.018672
52	6	0	-0.835068	10.744555	-0.530903
53	1	0	-2.081390	9.246603	-1.454759

54	6	0	0.364847	11.029808	0.121032
55	1	0	2.216799	10.176421	0.808934
56	1	0	-1.558073	11.540604	-0.690389
57	6	0	0.226313	-8.417461	-0.764536
58	6	0	-0.992145	-8.695882	-0.134352
59	6	0	1.136266	-9.457117	-0.963296
60	6	0	-1.269675	-9.980701	0.311218
61	1	0	-1.707181	-7.893216	0.018672
62	6	0	0.835068	-10.744555	-0.530903
63	1	0	2.081390	-9.246603	-1.454759
64	6	0	-0.364847	-11.029808	0.121032
65	1	0	-2.216799	-10.176421	0.808934
66	1	0	1.558073	-11.540604	-0.690389
67	6	0	-1.060681	6.953123	-2.523070
68	6	0	-2.179419	6.149391	-2.758933
69	6	0	-0.464559	7.619381	-3.596498
70	6	0	-2.671547	6.000845	-4.049604
71	1	0	-2.650009	5.637566	-1.924758
72	6	0	-0.980791	7.478194	-4.880093
73	1	0	0.398663	8.253065	-3.414215
74	6	0	-2.083632	6.659473	-5.132939
75	1	0	-3.539109	5.367609	-4.217598
76	1	0	-0.506929	8.005622	-5.704494
77	6	0	1.060681	-6.953123	-2.523070
78	6	0	2.179419	-6.149391	-2.758933
79	6	0	0.464559	-7.619381	-3.596498
80	6	0	2.671547	-6.000845	-4.049604
81	1	0	2.650009	-5.637566	-1.924758
82	6	0	0.980791	-7.478194	-4.880093
83	1	0	-0.398663	-8.253065	-3.414215
84	6	0	2.083632	-6.659473	-5.132939
85	1	0	3.539109	-5.367609	-4.217598
86	1	0	0.506929	-8.005622	-5.704494
87	6	0	3.123677	4.510688	-4.463763
88	6	0	2.361151	5.137302	-5.644662
89	6	0	3.356248	5.605152	-3.416370
90	6	0	4.488754	3.995150	-4.946992
91	1	0	2.193930	4.412385	-6.447222
92	1	0	1.386002	5.514877	-5.317394
93	1	0	2.934360	5.974302	-6.059937
94	1	0	3.925932	5.230993	-2.559093
95	1	0	3.927972	6.423896	-3.865511
96	1	0	2.408651	6.017814	-3.049458
97	1	0	5.082839	4.821713	-5.352989

98	1	0	5.046794	3.543127	-4.120628
99	1	0	4.379994	3.241625	-5.732962
100	6	0	-3.123677	-4.510688	-4.463763
101	6	0	-2.361151	-5.137302	-5.644662
102	6	0	-3.356248	-5.605152	-3.416370
103	6	0	-4.488754	-3.995150	-4.946992
104	1	0	-2.193930	-4.412385	-6.447222
105	1	0	-1.386002	-5.514877	-5.317394
106	1	0	-2.934360	-5.974302	-6.059937
107	1	0	-3.925932	-5.230993	-2.559093
108	1	0	-3.927972	-6.423896	-3.865511
109	1	0	-2.408651	-6.017814	-3.049458
110	1	0	-5.082839	-4.821713	-5.352989
111	1	0	-5.046794	-3.543127	-4.120628
112	1	0	-4.379994	-3.241625	-5.732962
113	7	0	0.176725	5.187059	3.170041
114	7	0	-0.176725	-5.187059	3.170041
115	6	0	0.495143	4.147720	4.057532
116	6	0	0.624531	4.436836	5.432066
117	6	0	0.682659	2.821668	3.599769
118	6	0	0.943539	3.435216	6.328419
119	1	0	0.475316	5.446238	5.795876
120	6	0	1.045765	1.851455	4.556100
121	6	0	1.173727	2.116317	5.909822
122	1	0	1.029998	3.683571	7.384130
123	1	0	1.264317	0.843926	4.214049
124	6	0	-0.495143	-4.147720	4.057532
125	6	0	-0.624531	-4.436836	5.432066
126	6	0	-0.682659	-2.821668	3.599769
127	6	0	-0.943539	-3.435216	6.328419
128	1	0	-0.475316	-5.446238	5.795876
129	6	0	-1.045765	-1.851455	4.556100
130	6	0	-1.173727	-2.116317	5.909822
131	1	0	-1.029998	-3.683571	7.384130
132	1	0	-1.264317	-0.843926	4.214049
133	5	0	0.486301	2.543709	2.093068
134	5	0	-0.486301	-2.543709	2.093068
135	6	0	0.000000	6.512098	3.689151
136	6	0	-1.244930	6.923649	4.151764
137	6	0	1.070239	7.404633	3.678997
138	6	0	-1.418052	8.231901	4.596472
139	1	0	-2.069116	6.216635	4.152080
140	6	0	0.884835	8.710162	4.116875
141	1	0	2.034912	7.066236	3.311778

142	6	0	-0.362575	9.145399	4.575477
143	1	0	-2.393075	8.551449	4.954643
144	1	0	1.719212	9.407047	4.098288
145	6	0	0.000000	-6.512098	3.689151
146	6	0	1.244930	-6.923649	4.151764
147	6	0	-1.070239	-7.404633	3.678997
148	6	0	1.418052	-8.231901	4.596472
149	1	0	2.069116	-6.216635	4.152080
150	6	0	-0.884835	-8.710162	4.116875
151	1	0	-2.034912	-7.066236	3.311778
152	6	0	0.362575	-9.145399	4.575477
153	1	0	2.393075	-8.551449	4.954643
154	1	0	-1.719212	-9.407047	4.098288
155	6	0	-1.564336	-1.041204	6.891966
156	1	0	-0.961522	-1.100473	7.804227
157	1	0	-1.423213	-0.045815	6.459577
158	1	0	-2.615533	-1.132342	7.188464
159	6	0	1.564336	1.041204	6.891966
160	1	0	0.961522	1.100473	7.804227
161	1	0	1.423213	0.045815	6.459577
162	1	0	2.615533	1.132342	7.188464
163	6	0	0.562427	-10.576662	5.004015
164	1	0	1.468732	-10.689487	5.604262
165	1	0	-0.285320	-10.936499	5.594276
166	1	0	0.657453	-11.231352	4.130426
167	6	0	-0.562427	10.576662	5.004015
168	1	0	-1.468732	10.689487	5.604262
169	1	0	0.285320	10.936499	5.594276
170	1	0	-0.657453	11.231352	4.130426
171	6	0	-0.691745	-12.424234	0.591698
172	1	0	-1.442577	-12.894715	-0.052867
173	1	0	0.196081	-13.062058	0.586608
174	1	0	-1.096599	-12.414605	1.608735
175	6	0	2.607596	-6.469187	-6.533290
176	1	0	2.393099	-7.339618	-7.159290
177	1	0	2.144644	-5.597498	-7.009472
178	1	0	3.689182	-6.307350	-6.533504
179	6	0	0.691745	12.424234	0.591698
180	1	0	1.442577	12.894715	-0.052867
181	1	0	-0.196081	13.062058	0.586608
182	1	0	1.096599	12.414605	1.608735
183	6	0	-2.607596	6.469187	-6.533290
184	1	0	-2.393099	7.339618	-7.159290
185	1	0	-2.144644	5.597498	-7.009472

186	1	0	-3.689182	6.307350	-6.533504

Supplementary Table 6. Cartesian Coordinate of ω-DABNA-PH (S_0, C_2 symmetry).
E(M062X/6-31G(d)) = –3866.859566 hartree

Center	Atomic	Atomic	Coordinates (Angstroms)		
Number	Number	Type	X	Y	Z
1	5	0	0.000000	0.000000	-1.994228
2	7	0	-0.558978	-2.353152	-0.474901
3	7	0	0.558978	2.353152	-0.474901
4	6	0	0.302338	1.179131	0.245493
5	6	0	0.000000	0.000000	-0.475601
6	6	0	-0.302338	-1.179131	0.245493
7	6	0	-0.351436	-1.191988	1.663571
8	6	0	0.000000	0.000000	2.304696
9	6	0	0.351436	1.191988	1.663571
10	1	0	0.000000	0.000000	3.389606
11	6	0	1.014346	2.296947	-1.819336
12	6	0	1.832177	3.328125	-2.300521
13	6	0	0.711630	1.197885	-2.647148
14	6	0	2.252772	3.366051	-3.622625
15	1	0	2.155719	4.115542	-1.631864
16	6	0	1.206913	1.223988	-3.966789
17	6	0	1.932162	2.289159	-4.466906
18	1	0	2.249657	2.301482	-5.505336
19	6	0	-1.014346	-2.296947	-1.819336
20	6	0	-0.711630	-1.197885	-2.647148
21	6	0	-1.832177	-3.328125	-2.300521
22	6	0	-1.206913	-1.223988	-3.966789
23	6	0	-2.252772	-3.366051	-3.622625
24	1	0	-2.155719	-4.115542	-1.631864
25	6	0	-1.932162	-2.289159	-4.466906
26	1	0	-2.249657	-2.301482	-5.505336
27	6	0	0.381970	3.621201	0.139188
28	6	0	0.571106	3.743854	1.530147
29	6	0	0.032480	4.726788	-0.630485
30	6	0	0.519132	5.031488	2.113325
31	6	0	0.000000	5.989206	-0.034258
32	1	0	-0.176387	4.638760	-1.689552

33	6	0	0.246450	6.159494	1.325707
34	1	0	0.214517	7.158299	1.742301
35	6	0	-0.381970	-3.621201	0.139188
36	6	0	-0.571106	-3.743854	1.530147
37	6	0	-0.032480	-4.726788	-0.630485
38	6	0	-0.519132	-5.031488	2.113325
39	6	0	0.000000	-5.989206	-0.034258
40	1	0	0.176387	-4.638760	-1.689552
41	6	0	-0.246450	-6.159494	1.325707
42	1	0	-0.214517	-7.158299	1.742301
43	1	0	1.016283	0.367248	-4.606681
44	1	0	-1.016283	-0.367248	-4.606681
45	7	0	-0.246130	7.130901	-0.842548
46	7	0	0.246130	-7.130901	-0.842548
47	6	0	0.708742	8.175473	-0.862970
48	6	0	2.065384	7.919891	-0.629368
49	6	0	0.299262	9.486922	-1.135741
50	6	0	2.989468	8.960123	-0.666719
51	1	0	2.391876	6.905231	-0.421943
52	6	0	1.232087	10.514719	-1.186912
53	1	0	-0.753359	9.685327	-1.312328
54	6	0	2.582509	10.260800	-0.950433
55	1	0	4.038263	8.742739	-0.487789
56	1	0	0.897792	11.525172	-1.401838
57	1	0	3.306923	11.067879	-0.985156
58	6	0	-0.708742	-8.175473	-0.862970
59	6	0	-2.065384	-7.919891	-0.629368
60	6	0	-0.299262	-9.486922	-1.135741
61	6	0	-2.989468	-8.960123	-0.666719
62	1	0	-2.391876	-6.905231	-0.421943
63	6	0	-1.232087	-10.514719	-1.186912
64	1	0	0.753359	-9.685327	-1.312328
65	6	0	-2.582509	-10.260800	-0.950433
66	1	0	-4.038263	-8.742739	-0.487789
67	1	0	-0.897792	-11.525172	-1.401838
68	1	0	-3.306923	-11.067879	-0.985156
69	6	0	-1.211378	7.034156	-1.877746
70	6	0	-2.468266	6.491302	-1.589237
71	6	0	-0.925758	7.448542	-3.182905
72	6	0	-3.420694	6.363655	-2.592658
73	1	0	-2.683015	6.169307	-0.574750
74	6	0	-1.892712	7.332668	-4.176490
75	1	0	0.053214	7.857621	-3.414488
76	6	0	-3.141946	6.789011	-3.890083

77	1	0	-4.390758	5.937867	-2.355315
78	1	0	-1.657442	7.656293	-5.185735
79	1	0	-3.890075	6.694202	-4.670262
80	6	0	1.211378	-7.034156	-1.877746
81	6	0	2.468266	-6.491302	-1.589237
82	6	0	0.925758	-7.448542	-3.182905
83	6	0	3.420694	-6.363655	-2.592658
84	1	0	2.683015	-6.169307	-0.574750
85	6	0	1.892712	-7.332668	-4.176490
86	1	0	-0.053214	-7.857621	-3.414488
87	6	0	3.141946	-6.789011	-3.890083
88	1	0	4.390758	-5.937867	-2.355315
89	1	0	1.657442	-7.656293	-5.185735
90	1	0	3.890075	-6.694202	-4.670262
91	7	0	0.727163	5.161561	3.486213
92	7	0	-0.727163	-5.161561	3.486213
93	6	0	1.072916	4.095462	4.332416
94	6	0	1.399551	4.370082	5.676728
95	6	0	1.101546	2.759807	3.857173
96	6	0	1.783017	3.345614	6.522587
97	1	0	1.366501	5.384968	6.053233
98	6	0	1.528690	1.758135	4.751589
99	6	0	1.866946	2.027348	6.064575
100	1	0	2.036843	3.581752	7.551947
101	1	0	1.619668	0.741742	4.381152
102	1	0	2.195513	1.233099	6.726564
103	6	0	-1.072916	-4.095462	4.332416
104	6	0	-1.399551	-4.370082	5.676728
105	6	0	-1.101546	-2.759807	3.857173
106	6	0	-1.783017	-3.345614	6.522587
107	1	0	-1.366501	-5.384968	6.053233
108	6	0	-1.528690	-1.758135	4.751589
109	6	0	-1.866946	-2.027348	6.064575
110	1	0	-2.036843	-3.581752	7.551947
111	1	0	-1.619668	-0.741742	4.381152
112	1	0	-2.195513	-1.233099	6.726564
113	5	0	0.712873	2.503885	2.388961
114	5	0	-0.712873	-2.503885	2.388961
115	6	0	0.635930	6.477965	4.049956
116	6	0	-0.566670	6.906068	4.605039
117	6	0	1.746300	7.318683	4.029362
118	6	0	-0.657097	8.185178	5.147365
119	1	0	-1.416548	6.230354	4.606511
120	6	0	1.650233	8.597975	4.569347

121	1	0	2.670866	6.960946	3.585991
122	6	0	0.449872	9.030852	5.129208
123	1	0	-1.593126	8.521857	5.581463
124	1	0	2.512496	9.256766	4.551793
125	1	0	0.376854	10.028769	5.549745
126	6	0	-0.635930	-6.477965	4.049956
127	6	0	0.566670	-6.906068	4.605039
128	6	0	-1.746300	-7.318683	4.029362
129	6	0	0.657097	-8.185178	5.147365
130	1	0	1.416548	-6.230354	4.606511
131	6	0	-1.650233	-8.597975	4.569347
132	1	0	-2.670866	-6.960946	3.585991
133	6	0	-0.449872	-9.030852	5.129208
134	1	0	1.593126	-8.521857	5.581463
135	1	0	-2.512496	-9.256766	4.551793
136	1	0	-0.376854	-10.028769	5.549745
137	6	0	-3.009468	-4.549890	-4.097724
138	6	0	-4.031590	-4.436875	-5.045706
139	6	0	-2.698992	-5.821460	-3.594755
140	6	0	-4.728089	-5.563007	-5.473704
141	1	0	-4.298756	-3.456051	-5.429168
142	6	0	-3.397854	-6.946642	-4.017013
143	1	0	-1.884693	-5.932793	-2.880833
144	6	0	-4.415715	-6.819773	-4.960376
145	1	0	-5.524877	-5.456902	-6.203685
146	1	0	-3.141651	-7.920418	-3.607512
147	1	0	-4.963154	-7.695852	-5.294151
148	6	0	3.009468	4.549890	-4.097724
149	6	0	4.031590	4.436875	-5.045706
150	6	0	2.698992	5.821460	-3.594755
151	6	0	4.728089	5.563007	-5.473704
152	1	0	4.298756	3.456051	-5.429168
153	6	0	3.397854	6.946642	-4.017013
154	1	0	1.884693	5.932793	-2.880833
155	6	0	4.415715	6.819773	-4.960376
156	1	0	5.524877	5.456902	-6.203685
157	1	0	3.141651	7.920418	-3.607512
158	1	0	4.963154	7.695852	-5.294151

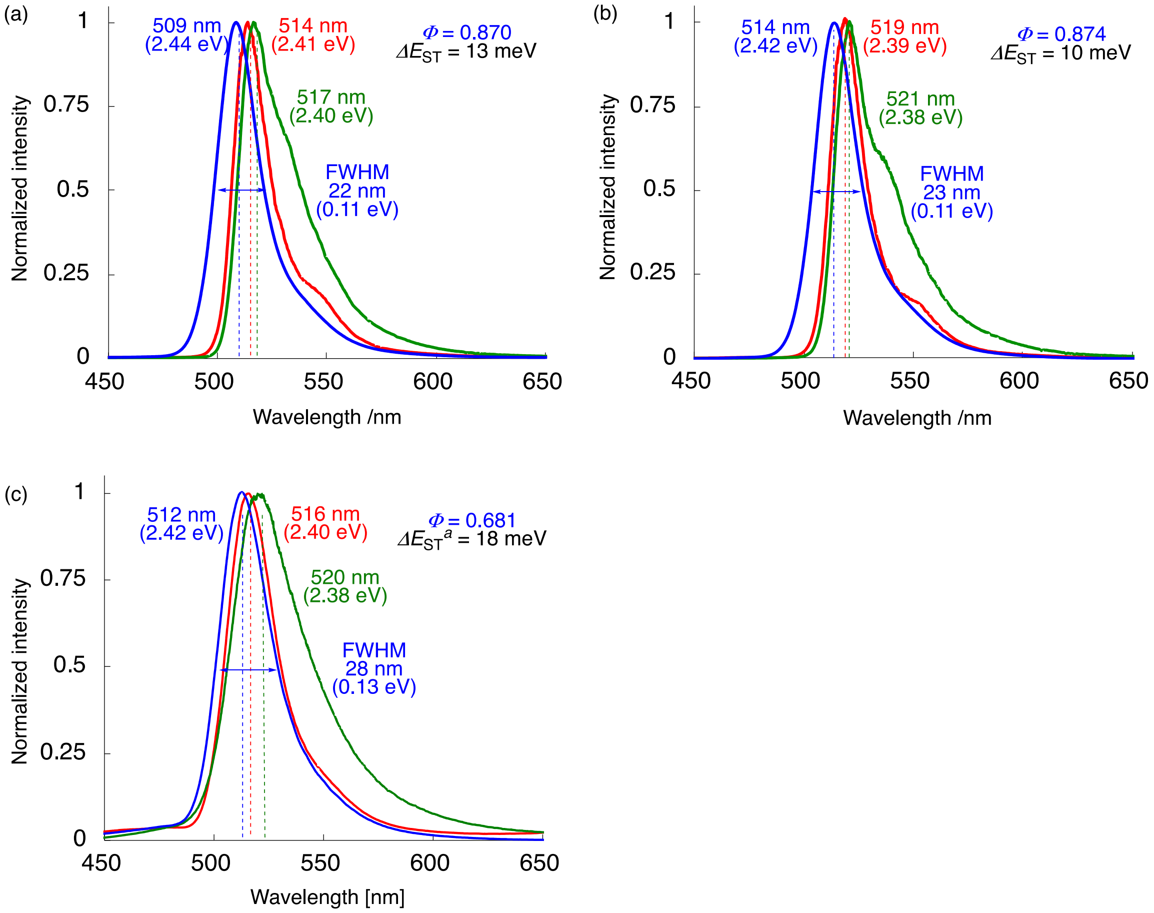

Supplementary Figure 20. Normalized fluorescence (blue, 298 K; red, 77K) and phosphorescence (green, 77 K, 25 msec delay) spectra of (a) **ω-DABNA**, (b) **ω-DABNA-M** and (c) **ω-DABNA-PH** with emission maxima (nm, eV), absolute fluorescence quantum yield ($\mathit{\Phi}$), full width at half maximum (nm, meV), and energy gap between S_1 and T_1 states estimated from emission maximum at 77 K ($\mathit{\Delta}E_{ST}$, meV) of 1 wt% PMMA films.

Supplementary Note 3

Estimation of rate constant for reverse intersystem crossing of **ω-DABNA**.

Rate constants (k_F, k_{IC}, k_{ISC}, and k_{RISC}) were determined from the measurements of quantum yields ($\mathit{\Phi}_F$ and $\mathit{\Phi}_{TADF}$) and lifetimes (τ_F, τ_{TADF}) and of the prompt (fluorescence) and delayed (TADF) components according to Adachi's method (equations 1–2[24], 3–5[25] and 6[26]).

$\mathit{\Phi}$ = 0.870

$\mathit{\Phi}_F$ = 0.819

$\mathit{\Phi}_{TADF}$ = 0.051

τ_F = 5.92 ns

τ_{TADF} = 8.95 μs

$k_p = 1.69 \times 10^8$ s^{-1}	$k_p = 1/\tau_F$	(1)
$k_d = 1.12 \times 10^5$ s^{-1}	$k_d = 1/\tau_{TADF}$	(2)
$k_F = 1.38 \times 10^8$ s^{-1}	$k_F = \Phi_F/\tau_F$	(3)
$k_{IC} = 2.07 \times 10^7$ s^{-1}	$\Phi = k_F/(k_F + k_{IC})$	(4)
$k_{ISC} = 9.86 \times 10^6$ s^{-1}	$\Phi_F = k_F/(k_F + k_{IC} + k_{ISC})$	(5)
$k_{RISC} = 1.19 \times 10^5$ s^{-1}	$k_{RISC} = k_p k_d/(k_p - k_{ISC})$	(6)

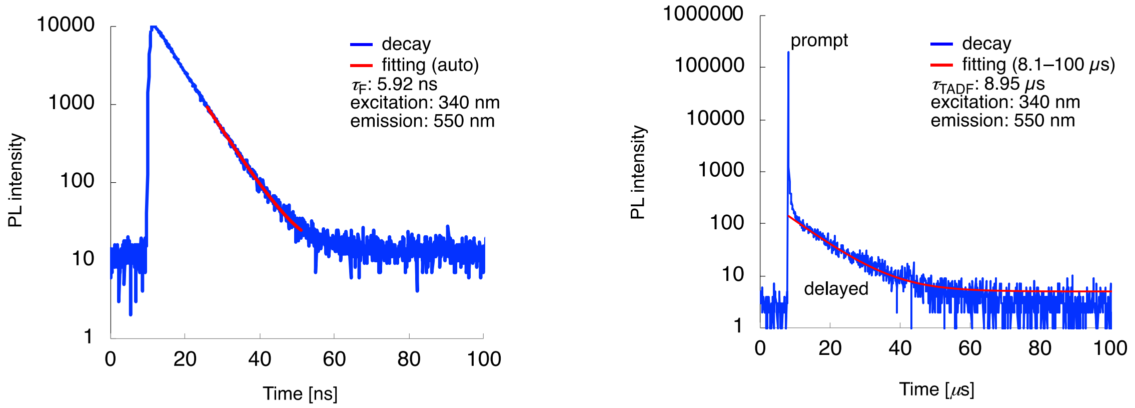

Supplementary Figure 21. Transient decay spectra of **ω-DABNA** in PMMA (1 wt%). A red curve is single exponential fitting data.

Supplementary Note 4

Estimation of rate constant for reverse intersystem crossing of **ω-DABNA-M**.

Φ = 0.874

Φ_F = 0.838

Φ_{TADF} = 0.036

τ_F = 5.60 ns

τ_{TADF} = 5.04 μs

$k_p = 1.79 \times 10^8$ s^{-1}	$k_p = 1/\tau_F$	(1)
$k_d = 1.98 \times 10^5$ s^{-1}	$k_d = 1/\tau_{TADF}$	(2)
$k_F = 1.50 \times 10^8$ s^{-1}	$k_F = \Phi_F/\tau_F$	(3)
$k_{IC} = 2.16 \times 10^7$ s^{-1}	$\Phi = k_F/(k_F + k_{IC})$	(4)
$k_{ISC} = 7.31 \times 10^6$ s^{-1}	$\Phi_F = k_F/(k_F + k_{IC} + k_{ISC})$	(5)
$k_{RISC} = 2.07 \times 10^5$ s^{-1}	$k_{RISC} = k_p k_d/(k_p - k_{ISC})$	(6)

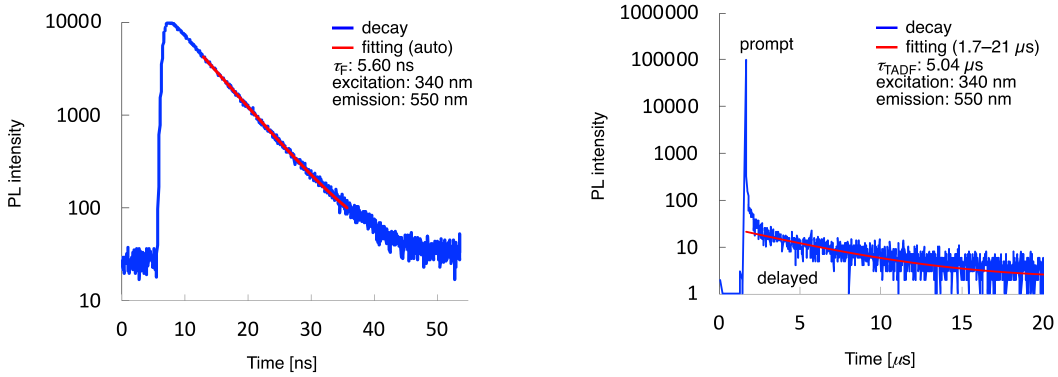

Supplementary Figure 22. Transient decay spectra of **ω-DABNA-M** in PMMA (1 wt%). A red curve is single exponential fitting data.

Supplementary Note 5

Estimation of rate constant for reverse intersystem crossing of **ω-DABNA-PH**.

$\Phi = 0.681$

$\Phi_F = 0.653$

$\Phi_{TADF} = 0.028$

$\tau_F = 4.62$ ns

$\tau_{TADF} = 4.70$ μs

$k_p = 2.16 \times 10^8\ s^{-1}$ $\quad$ $k_p = 1/\tau_F$ $\quad$ (1)

$k_d = 2.13 \times 10^5\ s^{-1}$ $\quad$ $k_d = 1/\tau_{TADF}$ $\quad$ (2)

$k_F = 1.41 \times 10^8\ s^{-1}$ $\quad$ $k_F = \Phi_F/\tau_F$ $\quad$ (3)

$k_{IC} = 6.62 \times 10^7\ s^{-1}$ $\quad$ $\Phi = k_F/(k_F + k_{IC})$ $\quad$ (4)

$k_{ISC} = 8.87 \times 10^6\ s^{-1}$ $\quad$ $\Phi_F = k_F/(k_F + k_{IC} + k_{ISC})$ $\quad$ (5)

$k_{RISC} = 2.22 \times 10^5\ s^{-1}$ $\quad$ $k_{RISC} = k_p k_d/(k_p - k_{ISC})$ $\quad$ (6)

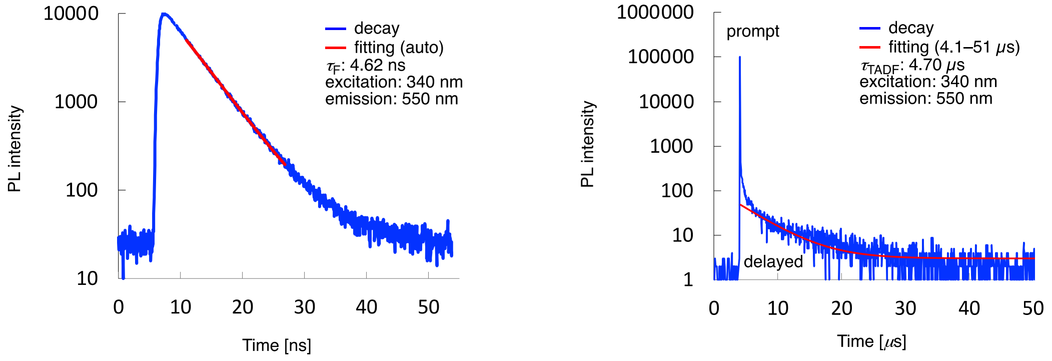

Supplementary Figure 23. Transient decay spectra of **ω-DABNA-PH** in PMMA (1 wt%). A red curve is single exponential fitting data.

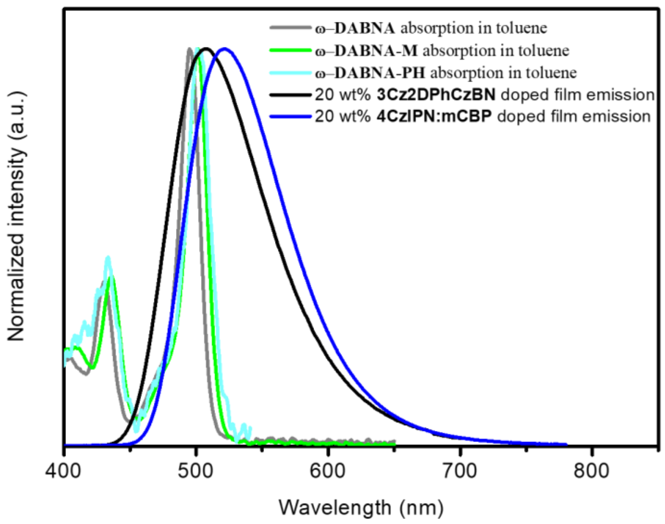

Supplementary Figure 24. Absorption spectra of MREs in toluene and emission spectra of sensitizer-only doped films.

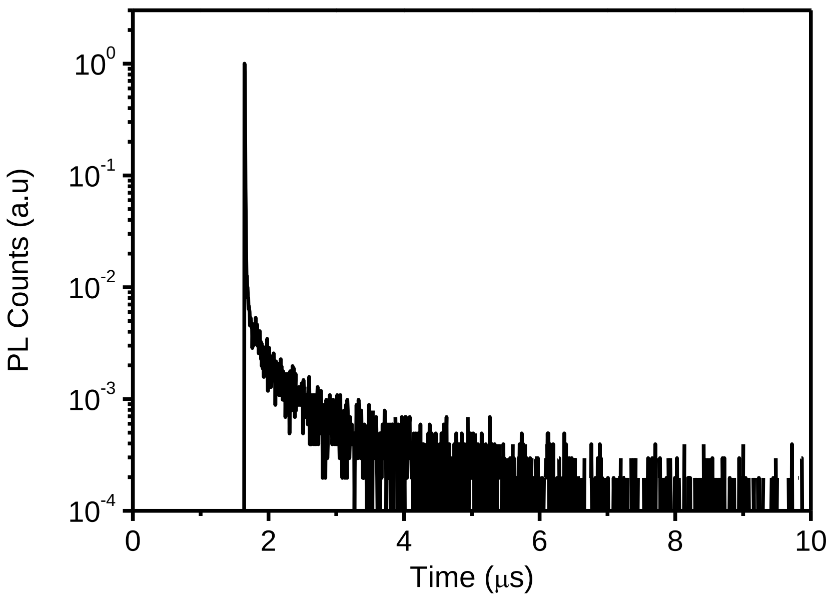

Supplementary Figure 25. PL decay curve of 1wt % **ω-DABNA**:20 wt% **3Cz2DPhCzBN** in mCBP film.

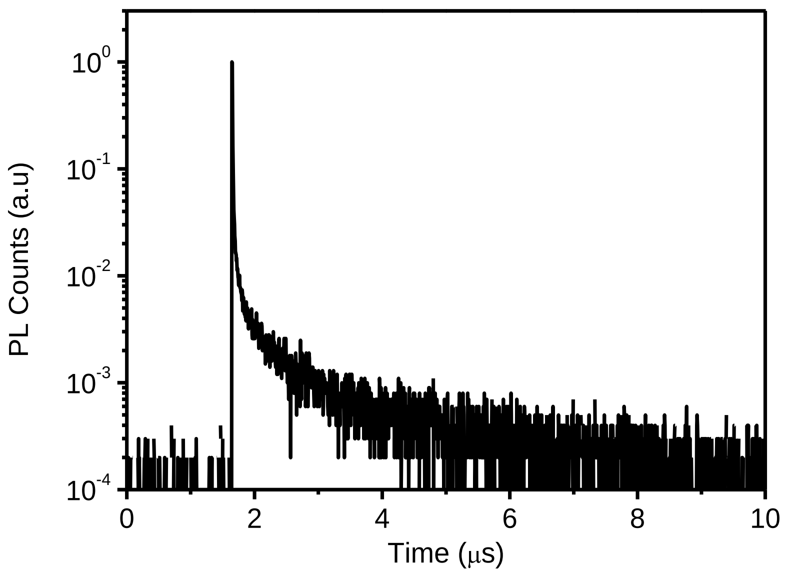

Supplementary Figure 26. PL decay curve of 1wt % **ω-DABNA-M**:20 wt% **3Cz2DPhCzBN** in mCBP film.

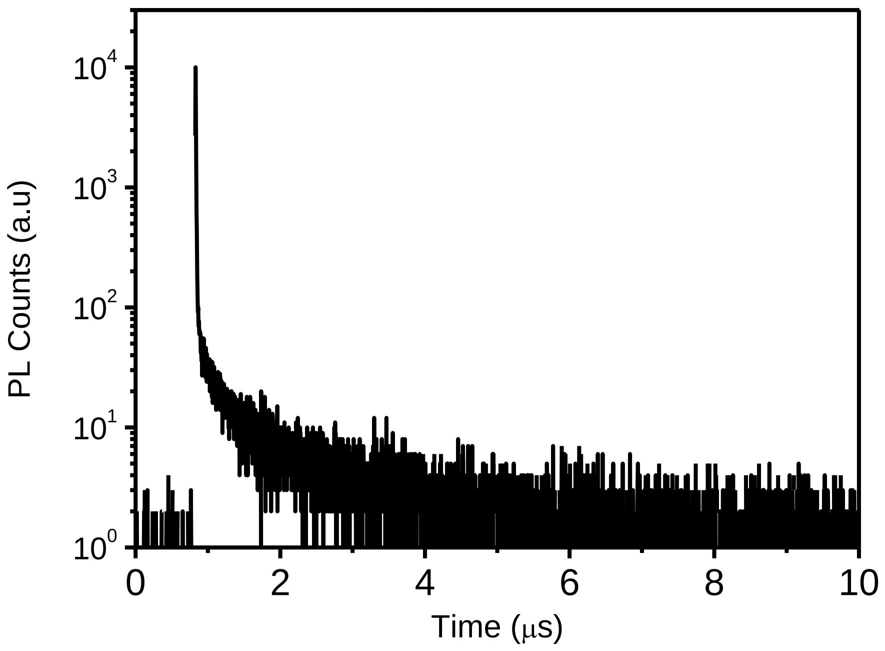

Supplementary Figure 27. PL decay curve of 1wt % **ω-DABNA-PH**:20 wt% **3Cz2DPhCzBN** in mCBP film.

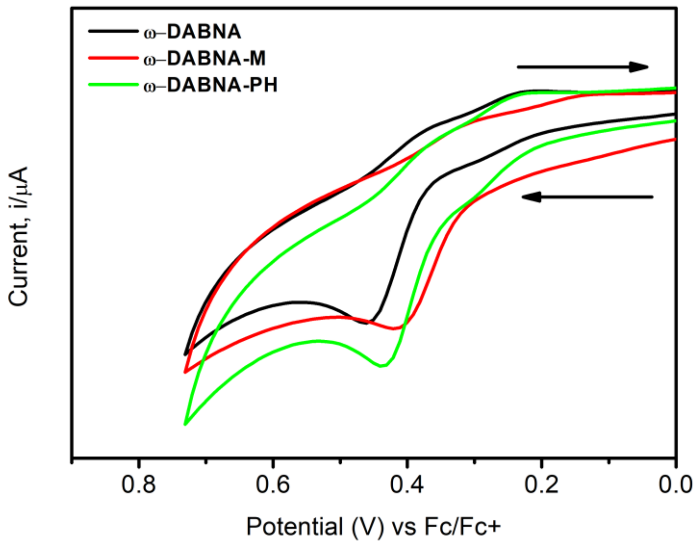

Supplementary Figure 28. Cyclic voltammogram of the oxidative scan of MREs in *N*,*N*-dimethylformamide. The HOMO energy levels of **ω-DABNA**, **ω-DABNA-M** and **ω-DABNA-PH** are determined to be –5.26, –5.21 and –5.24 eV, respectively.

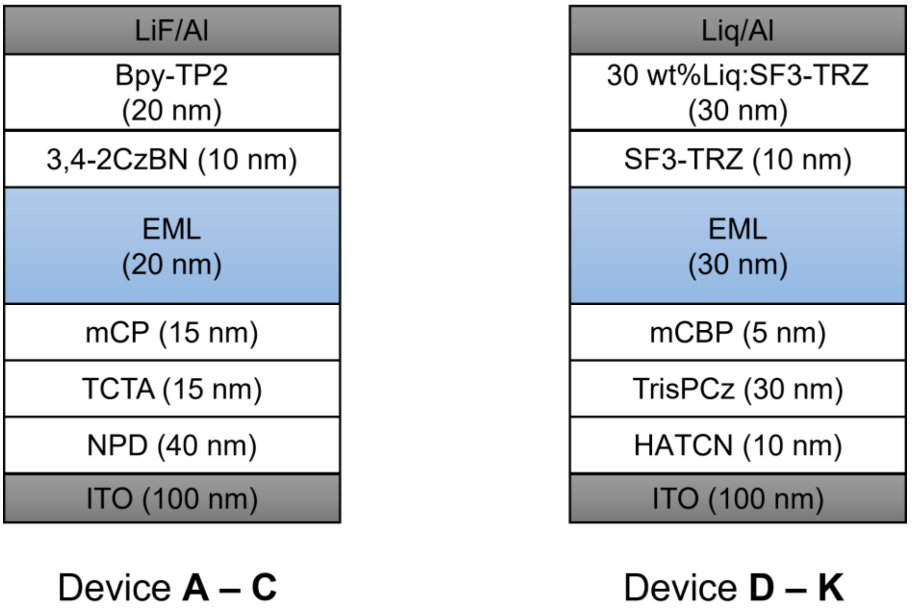

Supplementary Figure 29. Device structure of devices **A** – **K**.

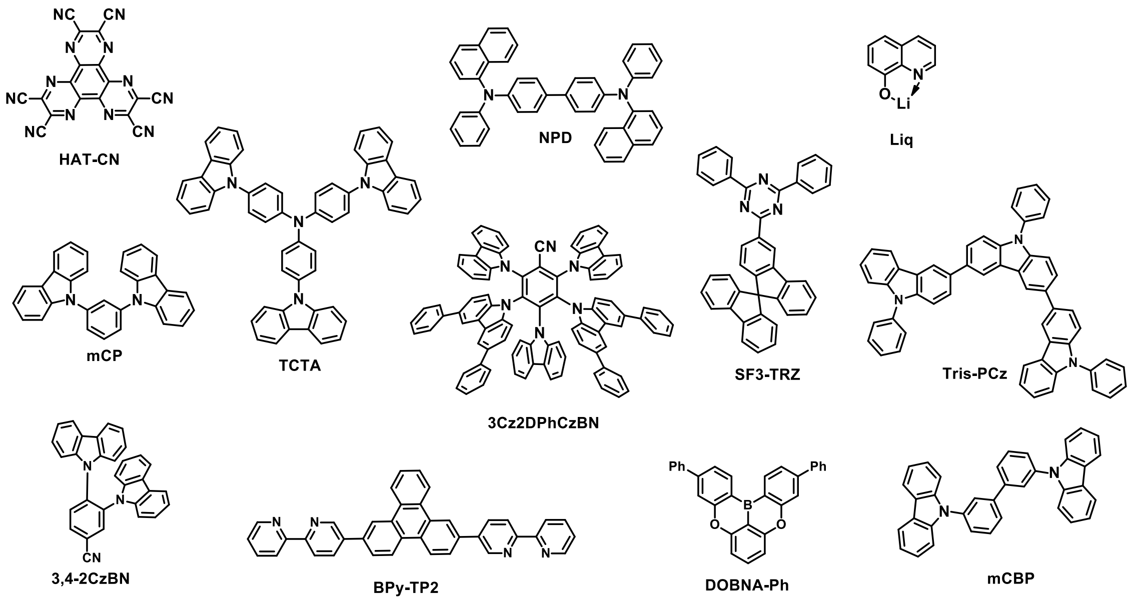

Supplementary Figure 30. Structures of materials used in devices **A** – **J**.

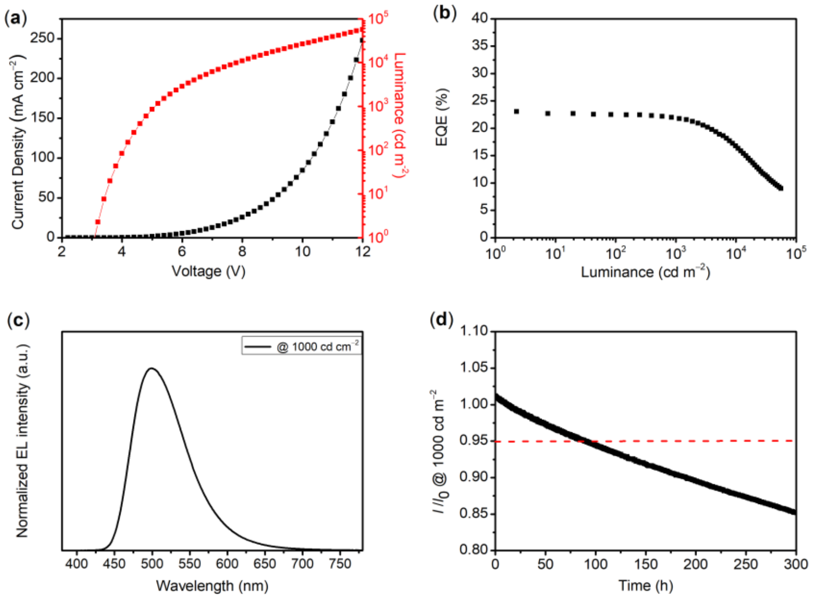

Supplementary Figure 31. Device performance of TADF OLED based on 20 % 3Cz2DPhCzBN:mCBP. (a) current density-voltage-luminance curve; (b) EQE versus luminance curve; (c) EL spectrum and (d) device stability (at an initial luminance of 1000 cd m^{-2}).

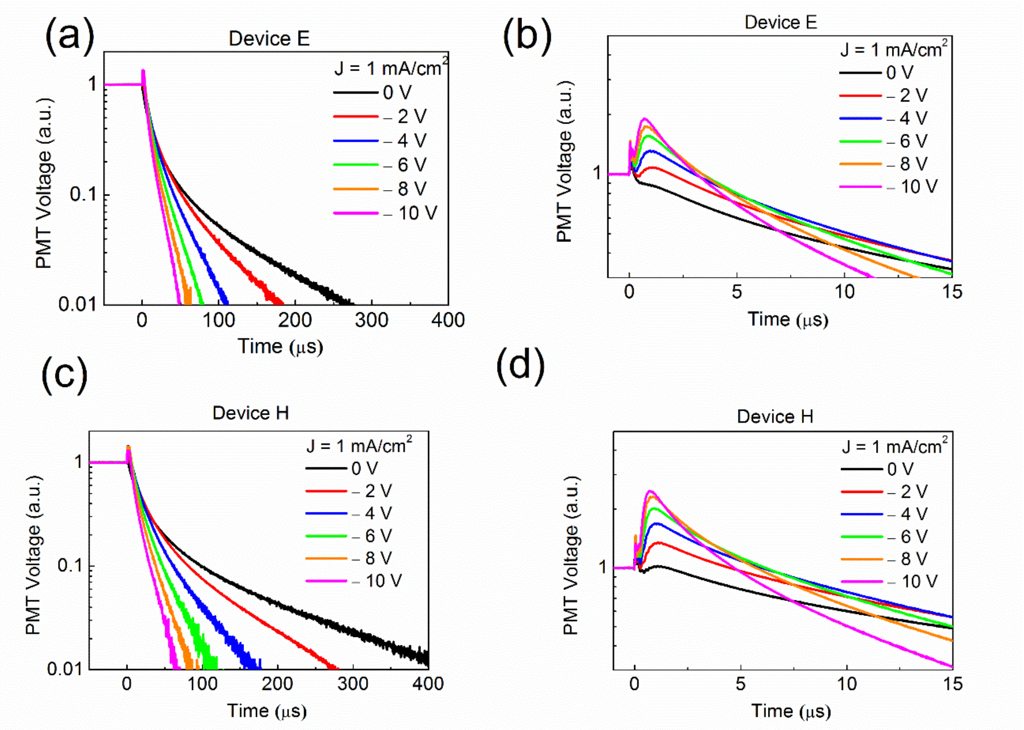

Supplementary Figure 32. **a** and **b**) Transient EL of devices of **E** (0.5 wt% **ω-DABNA-M**: 20 wt% 3Cz2DPhCzBN); **c** and **d**) Transient EL of devices **H** (1 wt% **ω-DABNA-M**: 20 wt% 3Cz2DPhCzBN).

Supplementary Table 7. Changes of $CIE_{x,y}$s with respect to luminance in device **A** – **K**.

Device	CIE (x, y)@ 1 cd m^{-2}	CIE (x, y)@ 100 cd m^{-2}	**CIE (x, y)@ 1000 cd m^{-2}**	CIE (x, y)@ 10000 cd m^{-2}
**A (reference)**	-	-	**(0.13, 0.73)**	-
**B**	-	-	**(0.15, 0.74)**	-
**C**	-	-	**(0.19, 0.74)**	-
**D**	(0.18, 0.60)	(0.15, 0.64)	**(0.15, 0.64)**	(0.15, 0.63)
**E**	(0.19, 0.60)	(0.18, 0.64)	**(0.18, 0.65)**	(0.17, 0.63)
**F**	(0.21, 0.62)	(0.20, 0.64)	**(0.20, 0.66)**	(0.20, 0.66)
**G**	(0.16, 0.66)	(0.15, 0.68)	**(0.14, 0.69)**	(0.14, 0.68)
**H**	(0.18, 0.66)	(0.17, 0.69)	**(0.17, 0.70)**	(0.17, 0.68)
**I**	(0.21, 0.66)	(0.21, 0.68)	**(0.21, 0.69)**	(0.20, 0.69)
**J**	(0.28, 0.64)	(0.27, 0.65)	**(0.27, 0.65)**	(0.27, 0.63)
**K**	(0.25, 0.68)	(0.24, 0.70)	**(0.24, 0.70)**	(0.24, 0.69)

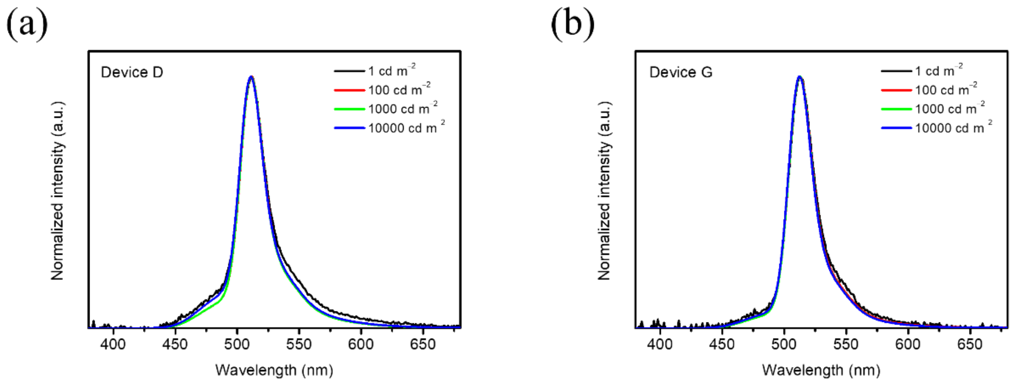

Supplementary Figure 33. Change of EL spectra with respective to luminance in **ω-DABNA**-based HF devices (a) **D** and (b) **G**.

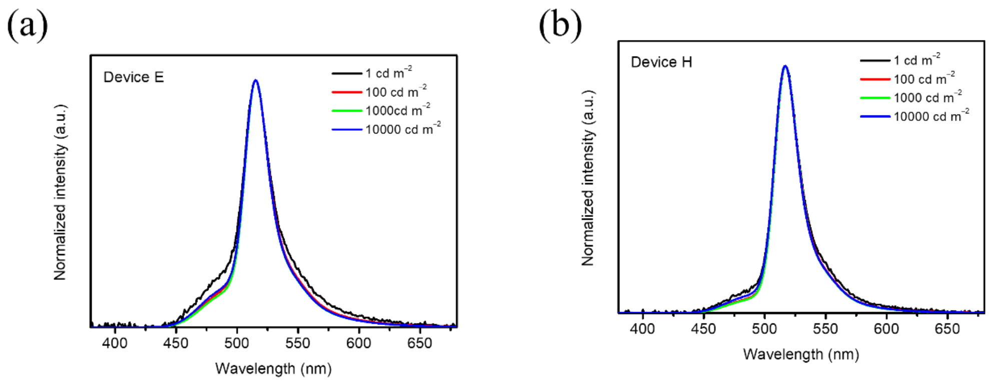

Supplementary Figure 34. Change of EL spectra with respective to luminance in **ω-DABNA-M**-based HF devices (a) **E** and (b) **H**.

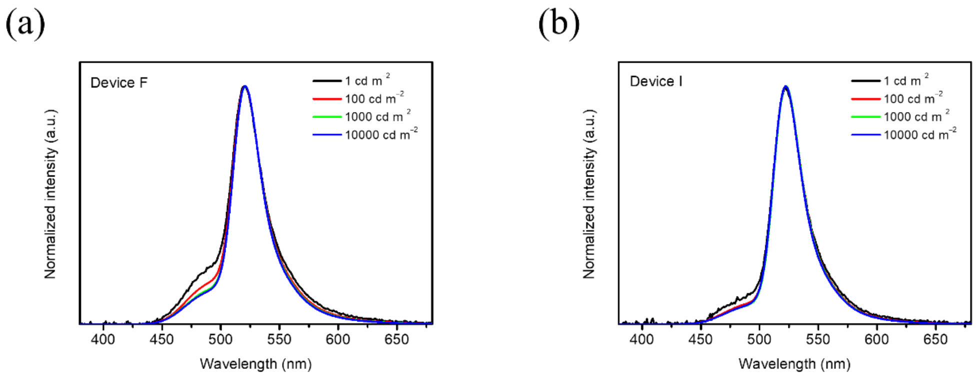

Supplementary Figure 35. Change of EL spectra with respective to luminance in **ω-DABNA-PH**-based HF devices (a) **F** and (b) **I**.

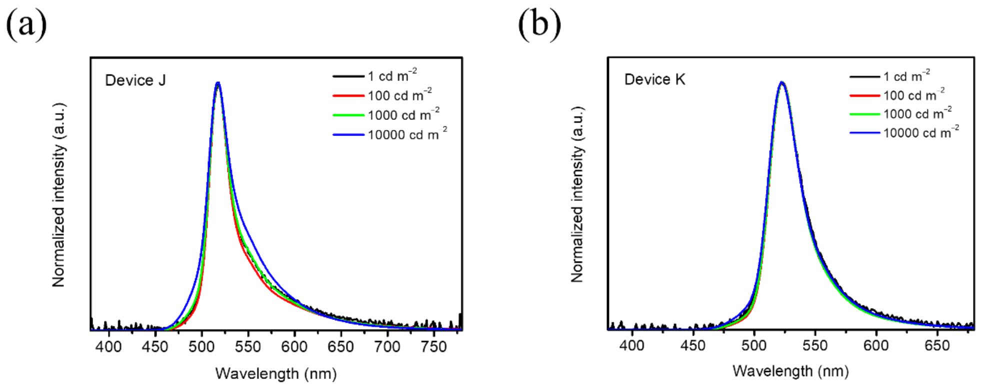

Supplementary Figure 36. Change of EL spectra with respective to luminance in 4CzIPN-based HF devices (a) **J** and (b) **K**.

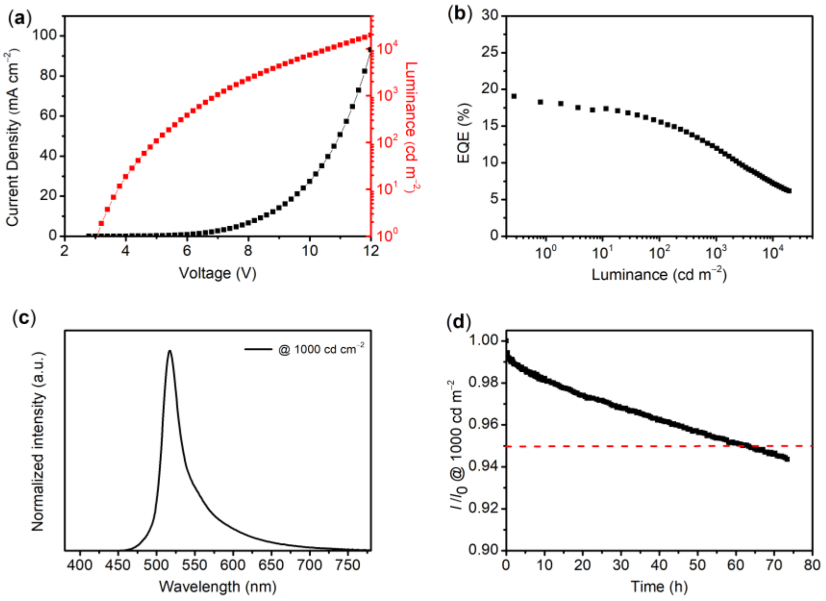

Supplementary Figure 37. Device characteristic of device **J** (a) $J-V$ and $L-V$ curves; (b) EQE versus luminance; (c) EL spectrum at 1000 cd m^{-2}; (d) device stability (at an initial luminance of 1000 cd m^{-2}).

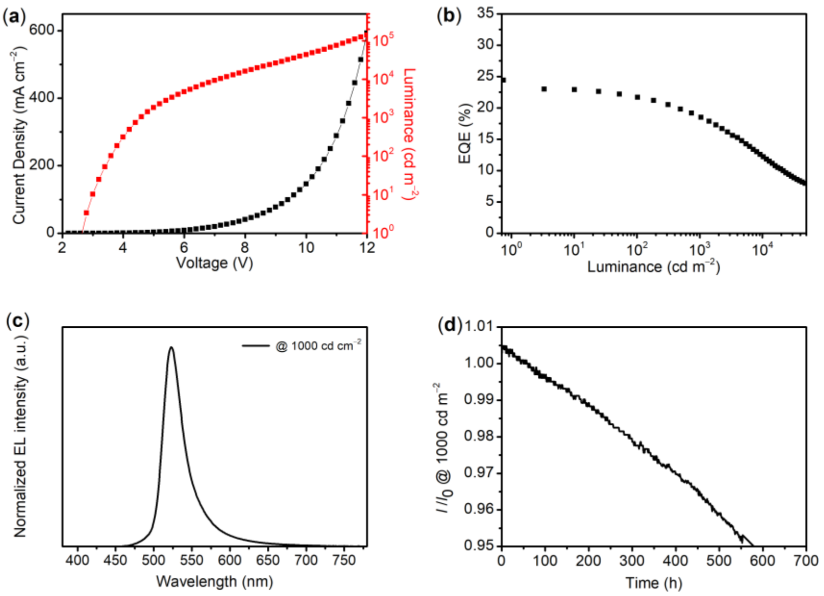

Supplementary Figure 38. Device characteristic of device **K** (a) $J-V$ and $L-V$ curves; (b) EQE versus luminance; (c) EL spectrum at 1000 cd m^{-2}; (d) device stability (at an initial luminance of 1000 cd m^{-2}).

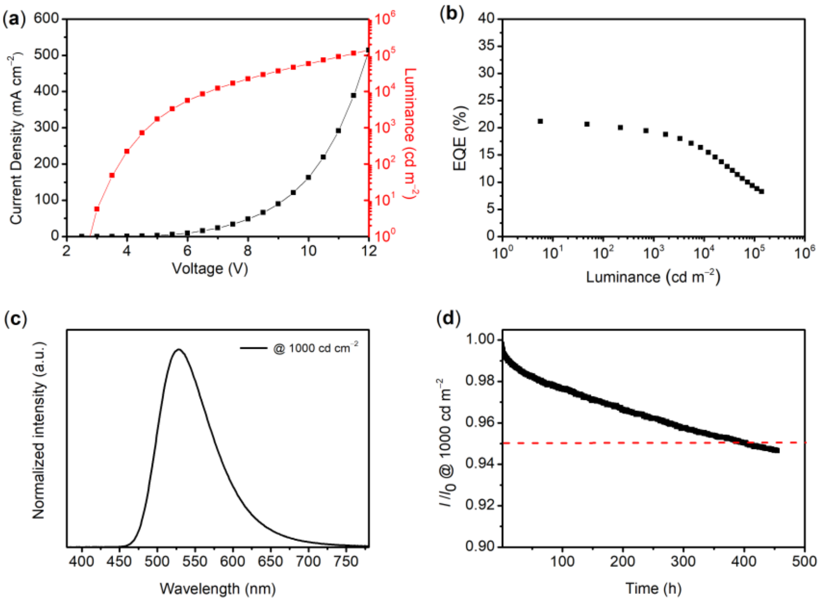

Supplementary Figure 39. Device performance of TADF OLED based on 20 % 4CzIPN:mCBP. ((a) $J-V$ and $L-V$ curves; (b) EQE versus luminance; (c) EL spectrum at 1000 cd m^{-2}; (d) device stability (at an initial luminance of 1000 cd m^{-2}).

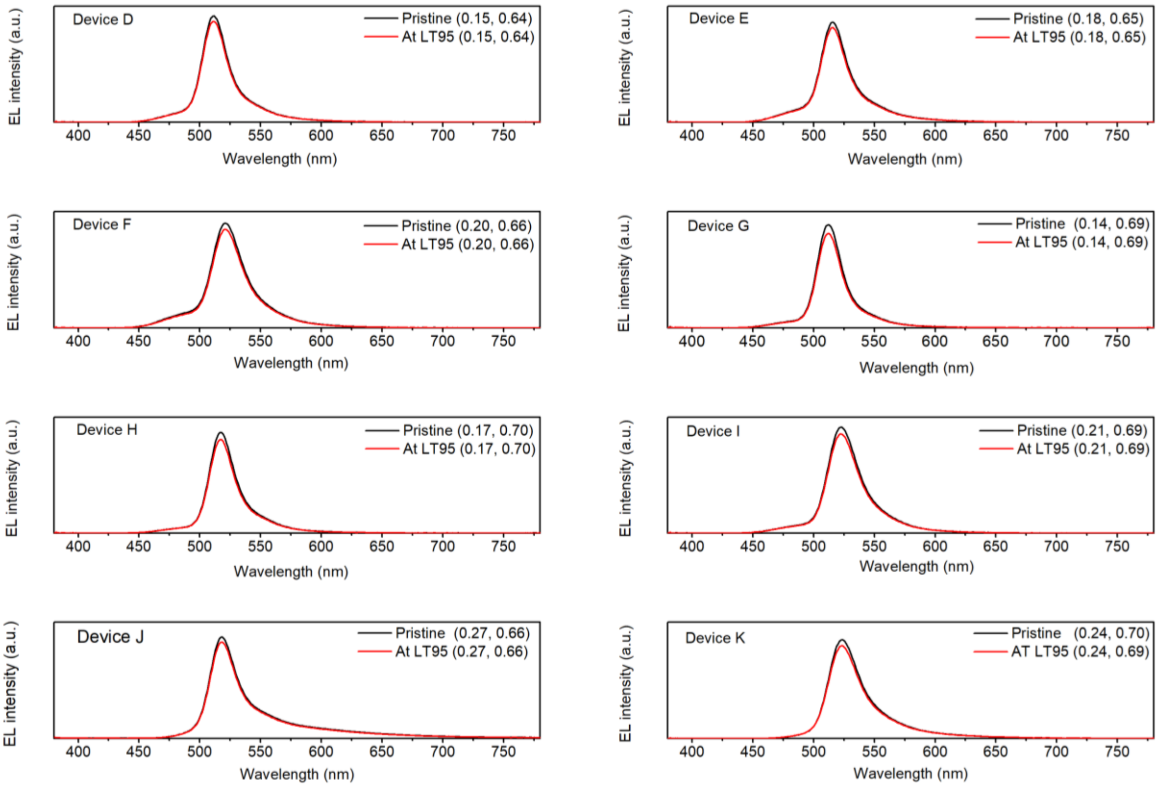

Supplementary Figure 40. Change of EL spectra with respective to time in various HF devices.

Supplementary References

1. Oda S. *et. al.* Development of Pure Green Thermally Activated Delayed Fluorescence Material by Cyano Substitution. *Adv. Mater*. **34**, 202201778 (2022).

2. Zhang Y. *et. al.* Multi-Resonance Induced Thermally Activated Delayed Fluorophores for Narrowband Green OLEDs. *Angew. Chem. Int. Ed*. **58**, 16912 –16917 (2019).

3. Zhang Y. *et. al.* Achieving Pure Green Electroluminescence with CIEy of 0.69 and EQE of 28.2% from an Aza-Fused Multi-Resonance Emitter. *Angew. Chem. Int. Ed*. **59**, 17499 –17503 (2020).

4. Xu Y. *et. al.* Constructing Charge-Transfer Excited States Based on Frontier Molecular Orbital Engineering: Narrowband Green Electroluminescence with High Color Purity and Efficiency. *Angew. Chem. Int. Ed.* **59**, 17442 –17446 (2020).

5. Xu Y. *et. al.* Highly Efficient Electroluminescence from Narrowband Green Circularly Polarized Multiple Resonance Thermally Activated Delayed Fluorescence Enantiomers. *Adv. Mater.* **33**, 2100652 (2021).

6. Jiang P. *et. al.* Simple Acridan-Based Multi-Resonance Structures Enable Highly Efficient Narrowband Green TADF Electroluminescence. *Adv. Opt. Mater.* **9**, 2100825 (2021).

7. Hua T. *et. al.* Heavy-atom Effect Promotes Multi-resonance Thermally Activated Delayed Fluorescence. *Chem. Eng. J.* **426**, 131169 (2021).

8. Qi Y. *et. al.* Peripheral Decoration of Multi-Resonance Molecules as a Versatile Approach for Simultaneous Long-Wavelength and Narrowband Emission. *Adv. Funct. Mater.* **31**, 2102017 (2021).

9. Liu G. *et. al.* Facile synthesis of multi-resonance ultra-pure-green TADF emitters based on bridged diarylamine derivatives for efficient OLEDs with narrow emission. *J. Mater. Chem. C* **9**, 8308–8313 (2021).

10. Ikeda N. *et. al.* Solution-Processable Pure Green Thermally Activated Delayed Fluorescence Emitter Based on the Multiple Resonance Effect. *Adv. Mater.* **32**, 2004072 (2020).

11. Oda S. *et. al.* Carbazole-Based DABNA Analogues as Highly Efficient Thermally Activated Delayed Fluorescence Materials for Narrowband Organic Light-Emitting Diodes. *Angew. Chem. Int. Ed*. **60**, 2882 –2886 (2021).

12. Yang M. *et. al.* Full-Color, Narrowband, and High-Efficiency Electroluminescence from Boron and Carbazole Embedded Polycyclic Heteroaromatics. *J. Am. Chem. Soc.* **142**, 19468−19472 (2020).

13. Wu X. *et. al.* Fabrication of Circularly Polarized MR-TADF Emitters with Asymmetrical Peripheral-Lock Enhancing Helical B/N-Doped Nanographenes. *Adv. Mater*. **34**, 2105080 (2022).

14. Xu Y. *et. al*. Constructing Organic Electroluminescent Material with Very High Color Purity and Efficiency Based on Polycyclization of Multiple Resonance Parent Core. *Angew. Chem. Int. Ed.,* **61**, e202204652 (2022).

15. Liu F. *et. al.* Highly Efficient Asymmetric Multiple Resonance Thermally Activated Delayed Fluorescence Emitter with EQE of 32.8% and Extremely Low Efficiency Roll-Off. *Angew. Chem. Int. Ed.* **61**, e202116927 (2022).

16. Yang Y. *et. al.* Chiral Multi-Resonance TADF Emitters Exhibiting Narrowband Circularly Polarized Electroluminescence with EQE of 37.2%. *Angew. Chem. Int. Ed.* **61**, e202202227 (2022).

17. Cai X. *et. al*. Achieving 37.1% Green Electroluminescent Efficiency and 0.09 eV Full Width at Half Maximum Based on a Ternary Boron-Oxygen-Nitrogen Embedded Polycyclic Aromatic System. *Angew. Chem. Int. Ed.* **61**, e2022003 (2022).

18. Zhang Y. *et. al.* Sterically Wrapped Multiple Resonance Fluorophors for Suppression of Concentration Quenching and Spectrum Broadening. *Angew. Chem. Int. Ed.* **61**, e202113206 (2022).

19. Uemura, S. *et. al*. Sequential Multiple Borylation Toward an Ultrapure Green Thermally Activated Delayed Fluorescence Material. *J. Am. Chem. Soc*. **145**, 1505–1511 (2023).

20. Kondo, M. Singlet-triplet energy gap of multiresonant molecular systems: A double hybrid time-dependent density functional theory study. *Chem. Phys. Lett.* **804**, 139895 (2022).

21. Gao, X. *et al.* Evaluation of Spin-Orbit Couplings with Linear-Response Time-Dependent Density Functional Methods. *J. Chem. Theory Comput.* **13**, 515 (2017).

22. Koseki, S. *et al.* MCSCF/6-31G(d,p) calculations of one-electron spin-orbit coupling constants in diatomic molecules. *J. Phys. Chem.* **96**, 10768 (1992).

23. Sun, Q. Libcint: An efficient general integral library for Gaussian basis functions. *J. Comput. Chem.* **36**, 1664 (2015).

24. Masui, K. *et. al*. Analysis of exciton annihilation in high-efficiency sky-blue organic light-emitting diodes with thermally activated delayed fluorescence. *Org. Electron.* **14**, 2721–2726 (2013).

25. Zhang, Q. *et. al*. Anthraquinone-Based Intramolecular Charge-Transfer Compounds: Computational Molecular Design, Thermally Activated Delayed Fluorescence, and Highly Efficient Red Electroluminescence. *J. Am. Chem. Soc*. **136**, 18070–18081 (2014).

26. Kaji, H. *et. al*. Purely organic electroluminescent material realizing 100% conversion from electricity to light. *Nat. Commun*. **6**, 8476 (2015).
